# Relationship of plasma biomarkers to digital cognitive tests in Alzheimer's disease

**DOI:** 10.1002/dad2.12590

**Published:** 2024-04-14

**Authors:** Sofia Toniolo, Sijia Zhao, Anna Scholcz, Benazir Amein, Akke Ganse‐Dumrath, Amanda J. Heslegrave, Sian Thompson, Sanjay Manohar, Henrik Zetterberg, Masud Husain

**Affiliations:** ^1^ Nuffield Department of Clinical Neurosciences University of Oxford Oxford UK; ^2^ Cognitive Disorders Clinic JR Hospital Oxford UK; ^3^ Department of Experimental Psychology University of Oxford Oxford UK; ^4^ UK Dementia Research Institute UCL London UK; ^5^ Department of Neurodegenerative Disease UCL Institute of Neurology London UK; ^6^ Institute of Neuroscience and Physiology University of Gothenburg Gothenburg Sweden; ^7^ Clinical Neurochemistry Laboratory Sahlgrenska University Hospital Mölndal Sweden; ^8^ Hong Kong Center for Neurodegenerative Diseases Hong Kong China; ^9^ Wisconsin Alzheimer's Disease Research Center University of Wisconsin School of Medicine and Public Health University of Wisconsin‐Madison Madison Wisconsin USA

**Keywords:** cognition, dementia, memory, online testing, phosphorylated tau

## Abstract

**INTRODUCTION:**

A major limitation in Alzheimer's disease (AD) research is the lack of the ability to measure cognitive performance at scale—robustly, remotely, and frequently. Currently, there are no established online digital platforms validated against plasma biomarkers of AD.

**METHODS:**

We used a novel web‐based platform that assessed different cognitive functions in AD patients (*N* = 46) and elderly controls (*N* = 53) who were also evaluated for plasma biomarkers (amyloid beta 42/40 ratio, phosphorylated tau ([p‐tau]181, glial fibrillary acidic protein, neurofilament light chain). Their cognitive performance was compared to a second, larger group of elderly controls (*N* = 352).

**RESULTS:**

Patients with AD were significantly impaired across all digital cognitive tests, with performance correlating with plasma biomarker levels, particularly p‐tau181. The combination of p‐tau181 and the single best‐performing digital test achieved high accuracy in group classification.

**DISCUSSION:**

These findings show how online testing can now be deployed in patients with AD to measure cognitive function effectively and related to blood biomarkers of the disease.

**Highlights:**

- This is the first study comparing online digital testing to plasma biomarkers.
- Alzheimer's disease patients and two independent cohorts of elderly controls were assessed.
- Cognitive performance correlated with plasma biomarkers, particularly phosphorylated tau (p‐tau)181.
- Glial fibrillary acidic protein and neurofilament light chain, and less so the amyloid beta 42/40 ratio, were also associated with performance.
- The best cognitive metric performed at par to p‐tau181 in group classification.

## 1 BACKGROUND

The advent of new disease‐modifying treatments for Alzheimer's disease (AD) has highlighted the need for sensitive cognitive tests to stratify those who might benefit from early interventions.^1^, ^2^ Traditional face‐to‐face neuropsychological assessments can detect changes only several years after pathological accumulation of amyloid and tau, a factor that might have led to past clinical trial failures.^3^ Digital cognitive metrics capture subtle signs of impairment that cannot be measured by standard clinical tests and might be particularly valuable in early phases of the disease when cognitive impairment is at subthreshold levels on current scales.^4^ Screening for AD, recruitment, and longitudinal follow‐up in clinical trials could all be transformed if cognitive testing were to be conducted robustly, remotely, and frequently.^5^

Recognized biomarkers for AD diagnosis are currently divided into three main categories according to the ATN (amyloid, tau, and neurodegeneration) staging system^6^; amyloid markers (reduced amyloid beta [Aβ]42 or Aβ42/Aβ40 ratio in the cerebrospinal fluid [CSF], or positive amyloid positron emission tomography [PET]), tau (increased phosphorylated tau [p‐tau]181 in the CSF or positive tau PET), and neurodegeneration (atrophy on magnetic resonance imaging [MRI], positive 18F‐fluorodeoxyglucose [FDG] PET, and increased total tau [t‐tau] in the CSF).

However, work on plasma biomarkers for AD has advanced rapidly. Regarding amyloid, plasma Aβ42/40 ratio has been found to correlate well with its CSF levels and with amyloid PET findings, and its reduction is associated with cognitive decline in cognitively unimpaired individuals, and people with subjective cognitive impairment (SCI) and mild cognitive impairment (MCI).^6^, ^7^, ^8^, ^9^, ^10^

Plasma p‐tau181 has been emerging as an even more specific and sensitive biomarker for AD.^10^, ^11^, ^12^ It correlates well with its levels in the CSF,^10^ and is associated with both amyloid and tau PET positivity.^10^, ^12^ It is elevated in amyloid‐positive individuals, even in the pre‐symptomatic stage, while it is not increased in several clinical mimics of AD.^10^, ^13^

Plasma glial fibrillary acidic protein (GFAP), which is a marker of neuroinflammation and reflects astrocytosis, is also associated with amyloid deposition in healthy controls, and SCI, MCI, and AD dementia patients.^14^, ^15^, ^16^, ^17^ Some evidence suggests that it is better than the Aβ42/40 ratio in predicting amyloid PET positivity.^14^, ^17^ However, raised GFAP levels are not specific to AD, and are also increased in many other neurological diseases.^18^ Similarly to GFAP, another plasma biomarker that is altered across several neurological disorders is neurofilament light chain (NfL). High baseline levels of NfL, an index of the rate of axonal injury, are strongly linked to markers of neurodegeneration such as CSF t‐tau, MRI atrophy, and FDG‐PET hypometabolism.^19^, ^20^, ^21^

Cognitive performance may be the single most important factor to increase the diagnostic accuracy of plasma biomarkers in AD, compared to other measures including MRI imaging and apolipoprotein E status.^22^ Although some studies have examined the relationship between plasma biomarkers and cognition, to our knowledge, the cognitive tests used were not digital online measures. For example, baseline levels and longitudinal increases in plasma p‐tau181 are associated with a decline in standard tests of cognition such as the Mini‐Mental State Examination (MMSE).^11^, ^16^, ^23^, ^24^, ^25^, ^26^ The increase of another p‐tau isoform, plasma p‐tau217, was found to correlate with worsening cognition on the MMSE and modified Preclinical Alzheimer Cognitive Composite (mPACC)^27^ in cognitively unimpaired and MCI participants. High GFAP and NfL levels have also been linked to worse cognitive performance on standard tests of cognition,^17^, ^28^, ^29^ to a decline in cognition over time,^19^, ^21^, ^30^, ^31^ and clinical conversion to AD.^15^ However, these changes do not seem to be AD specific.^32^

A head‐to‐head study comparing different plasma biomarkers in cognitively unimpaired individuals with positive amyloid status^33^ found that p‐tau217, p‐tau181, and GFAP were associated with cognitive decline (on the mPACC and MMSE), while p‐tau231 and NfL were not. p‐tau217 was the strongest individual predictor of cognitive impairment. However, standard neuropsychological tests such as the mPACC and the MMSE still require a dedicated face‐to‐face appointment, which limits their use for large‐scale population screening. While the associations with standard cognitive metrics might shed light on the ability of specific biomarkers to capture overall global cognitive impairment using standard pen‐and‐paper tests, research is needed on whether this applies to digital tests as well. Currently, to the best of our knowledge, there are no published studies that have reported on the association between plasma biomarkers and performance on a panel of digital cognitive tests.

Here, we investigated whether plasma biomarkers (Aβ42/40 ratio, p‐tau181, GFAP, and NfL) are associated with several digital online cognitive metrics, measuring visual short‐term memory, long‐term memory, visuospatial copying, executive function, and processing speed in a cohort of patients with AD and two samples of elderly healthy controls, one of which also underwent blood collection for plasma biomarker measurement. While cognitive impairment has historically been a key feature of all previous diagnostic criteria for AD,^7^, ^8^ according to the ATN criteria its presence is not necessary to support AD diagnosis. Conversely, AD as defined by the in vivo detection of the accumulation of amyloid and tau can occur even in asymptomatic individuals. Here, in a real‐world cohort of patients, we used the standard clinical criteria for diagnosis of probable AD,^34^ which do not require CSF or PET imaging evidence of ATN positivity, and examined the relationship of clinical classification to performance on our digital cognitive tests and plasma biomarkers of AD.

## 2 METHODS

### 2.1 Ethics

The study was performed in accordance with the ethical standards as laid down in the 1964 Declaration of Helsinki and its later amendments. Ethical approval was granted by the University of Oxford ethics committee (IRAS ID: 248379, Ethics Approval Reference: 18/SC/0448). All participants gave written informed consent prior to the start of the study.

### 2.2 Participants

Forty‐six patients with AD and 53 elderly healthy controls (EHC) were recruited, respectively, from the Cognitive Disorders Clinic (AD) at the John Radcliffe Hospital in Oxford, UK, or open day events (EHC). Patients with AD had a progressive, multidomain, largely amnestic cognitive impairment and underwent MRI and FDG‐PET imaging, the results of which were in keeping with a clinical diagnosis of AD (temporo‐parietal atrophy and hypometabolism),^34^ but did not have ATN confirmation prior to enrollment in the study. Two patients in the AD group scored above 88/100 on the Addenbrooke's Cognitive Examination‐III (ACE),^35^ which is considered in the normal range. Three patients in the AD group scored in the MCI range (ACE scores between 87 and 82), while all the other patients scored < 82, which is compatible with a neuropsychological diagnosis of dementia. Elderly healthy controls were >50 years old, had no psychiatric or neurological illness, were not on regular psychoactive drugs, and all scored above the cut‐off for normality (88/100 total ACE score). They also underwent brain MRI imaging, and only participants with a normal MRI scan, reviewed by two independent senior neurologists, were included in the study. The groups were not statistically different in age, sex, or education level (Table 1).

**RESEARCH IN CONTEXT**

1. **Systematic Review**: The literature was reviewed using PubMed for articles regarding blood‐based biomarkers and digital cognitive testing. While data comparing the performance of plasma biomarkers and brief standard cognitive tests were widely available, studies using digital platforms were lacking. Conversely, digital tests’ performance has been validated against markers of Alzheimer's disease (AD) in cerebrospinal fluid or positron emission tomography imaging, but not plasma biomarkers. This study is the first to combine these two methods.
2. **Interpretation**: The digital metrics used here were associated with plasma biomarkers, with phosphorylated tau181 and amyloid beta 42/40 ratios showing, respectively, the highest and lowest association with cognition. A digital platform validated against AD plasma biomarkers provides an important step forward for future large‐scale deployment.
3. **Future Directions**: Digital and plasma biomarkers will be essential for population screening, clinical trials recruitment, and drug monitoring, being widely accessible and cost effective. Further studies are needed to validate these results in larger and more diverse cohorts.

**TABLE 1:** Demographics, plasma biomarkers, standard cognitive metrics, and questionnaire‐derived apathy and depression scores.

					AD vs. EHC 1	
Dimensions	Metrics	AD (*n* = 46)	EHC 1 (*n* = 53)	EHC 2 (*n* = 352)	*P* value	Rank‐biserial correlation
Demographics	Age	68.3 (10.2)	68.6 (7.0)	59.9 (8.5)	n.s.	
	Sex (M/F)	23/23	24/29	177/175	n.s.	
	Education	14.5 (3.5)	15.8 (3.1)	15.0 (2.1)	n.s.	
Plasma biomarkers	p‐tau181 (pg/mL)	5.4 (3.4)	2.6 (1.3)	Not applicable	* < 0.001	−0.70
	GFAP (pg/mL)	224.7 (118)	111.8 (57.9)	Not applicable	* < 0.001	−0.69
	NfL (pg/mL)	29.7 (19)	17.1 (11)	Not applicable	* < 0.001	−0.58
	Aβ42/40 ratio	0.059 (0.01)	0.067 (0.01)	Not applicable	* < 0.001	0.53
	Aβ42 (pg/mL)	6.6 (1.5)	7.1 (1.5)	Not applicable	0.06	0.22
	Aβ40 (pg/mL)	115.2 (25.7)	108.4 (19.9)	Not applicable	n.s.	−0.13
ACE	Total score	63.5 (20.0)	97.4 (2.0)	Not applicable	* < 0.001	0.93
	Attention	12.0 (4.4)	17.2 (0.3)	Not applicable	* < 0.001	0.88
	Memory	11.7 (6.1)	25.0 (1.1)	Not applicable	* < 0.001	0.95
	Fluency	7.4 (3.6)	13.2 (0.9)	Not applicable	* < 0.001	0.89
	Language	21.0 (4.9)	25.6 (0.7)	Not applicable	* < 0.001	0.78
	Visuospatial	11.9 (4.2)	15.8 (0.3)	Not applicable	* < 0.001	0.79
Questionnaires	AMI	1.5 (0.3)	1.2 (0.4)	1.5 (0.4)	*0.002	−0.40
	GDS	6.9 (1.9)	4.9 (1.1)	5.7 (2.0)	* < 0.001	−0.64

*Notes*: All metrics are reported in group mean and standard deviation. * Indicates *P* values below <0.001, n.s. means not significant. The effect size for group comparison is the rank‐biserial correlation coefficient.

Abbreviations: Aβ, amyloid beta; ACE, The Addenbrooke's Cognitive Examination‐III; AD, Alzheimer's disease; AMI, Apathy Motivation Index; EHC, elderly healthy control; GDS, Geriatric Depression Scale; GFAP, glial fibrillary acidic protein; NfL, neurofilament light chain; p‐tau, phosphorylated tau.

**FIGURE 1:**
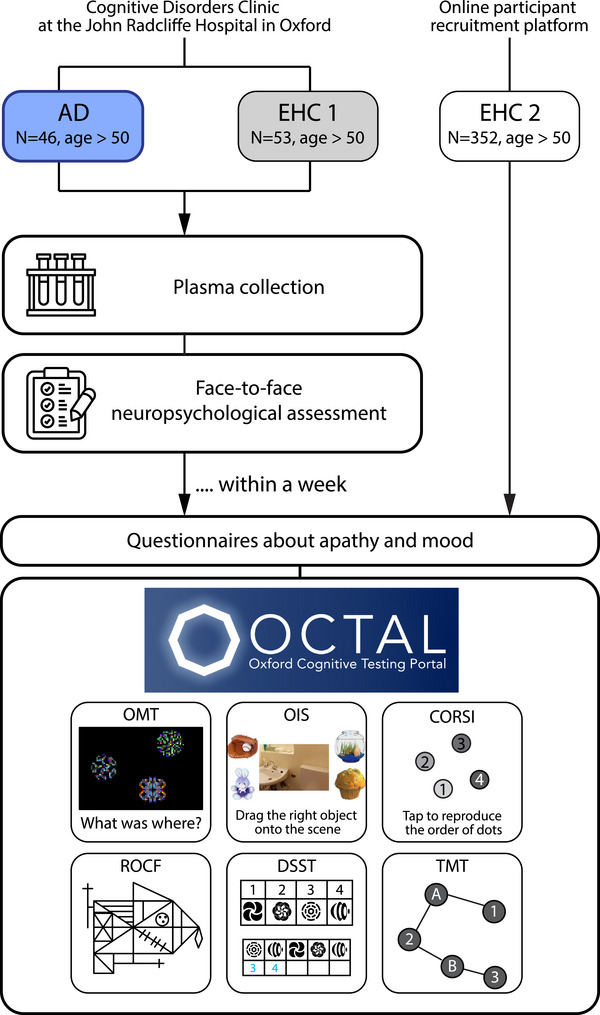
Study schematic. AD, Alzheimer's disease; CORSI, Corsi Tapping Task; DSST, Digit Symbol Substitution Test; EHC, elderly healthy control; OIS, Object‐in‐Scene; OMT, Oxford Memory Test; ROCF, Rey–Osterrieth Complex Figure; TMT, Trail Making Test.

Participants underwent blood collection, face‐to‐face standard cognitive, and online remote digital cognitive testing, and self‐reported motivation and mood indices were collected (see Figure 1 for study flow).

Because human behavior sampled for convenience only across university populations may be WEIRD (Western, Educated, Industrialized, Rich, and Democratic),^36^ the healthy controls recruited through the University (EHC1) may not necessarily be representative of the general population. Therefore, we recruited 356 healthy participants > 50 years old online through the Prolific participant recruitment platform (prolific.co) as a second normative group (group EHC2, see Table 1 for basic demographics). All participants were blinded with respect to the aim of this study which was advertised as “a brain game” testing how well people could perform. They had normal or corrected‐to‐normal vision acuity and no color blindness. All were residents of the UK, had English as their first language, and self‐reported to be neurologically healthy. Four participants from this group were excluded due to failing multiple attention checks (see section 2.7: Attention checks), leaving this group with 352 valid participants. This EHC2 group was well representative of ethnic groups and subjective socioeconomic status of the general public of the UK (see Methods S1 in supporting information for more details). To account for the effect of age on cognitive metrics, the cognitive performance of all participants was transformed into *z* score based on EHC2 group (see Table 2 and S1 in supporting information).

**TABLE 2:** Digital cognitive metrics from OCTAL.

Digital Cognitive Tasks	Metrics	AD (*n* = 46)	EHC 1 (*n* = 53)	EHC 2 (*n* = 352)	AD vs. EHC 1	AD vs. EHC 2	EHC 1 vs. EHC 2
OMT	Identification Accuracy (z)	−1.3 (1.9)	0.6 (1.0)	−0.2 (1.4)	*r* = 0.59, *P* < 0.0001	*r* = 0.38, *P* = 0.0027	*r* = −0.37, *P* < 0.0001
	Location Error (z)	4.4 (3.4)	0.9 (1.7)	0.2 (1.4)	*r* = −0.64, *P* < 0.0001	*r* = −0.77, *P* < 0.0001	*r* = −0.33, *P* = 0.0002
	Target Detection Rate (z)	−1.3 (1.6)	0.7 (0.9)	−0.1 (1.2)	*r* = 0.71, *P* < 0.0001	*r* = 0.47, *P* = 0.0002	*r* = −0.46, *P* < 0.0001
	Misbinding Error Rate(z)	36.9 (11.8)	21.7 (7.9)	28.1 (10.2)	*r* = −0.68, *P* < 0.0001	*r* = −0.47, *P* = 0.0001	*r* = 0.42, *P* < 0.0001
OIS	Object Identification Accuracy—Delayed Recall (z)	−2.5 (1.3)	0.0 (1.2)	−0.1 (1.1)	*r* = 0.82, *P* < 0.0001	*r* = 0.82, *P* < 0.0001	*r* = −0.07, n.s.
	Location Error—Delayed Recall (z)	4.0 (3.5)	0.2 (1.0)	0.3 (1.5)	*r* = −0.70, *P* < 0.0001	*r* = −0.68, *P* < 0.0001	*r* = −0.05, n.s.
ROCF	Copy Raw Score (%)	78.5 (30.2)	98.3 (3.1)	97.3 (4.9)	*r* = 0.66, *P* < 0.0001	*r* = 0.57, *P* < 0.0001	*r* = −0.11, n.s.
	Copy Score (z)	−5.5 (8.8)	0.1 (0.9)	−0.3 (1.6)	*r* = 0.67, *P* < 0.0001	*r* = 0.57, *P* < 0.0001	*r* = −0.06, n.s.
	Immediate Recall Raw Score (%)	43.1 (22.8)	89.9 (11.8)	86.1 (13.2)	*r* = 0.92, *P* < 0.0001	*r* = 0.88, *P* < 0.0001	*r* = −0.17, n.s.
	Immediate Recall Score (z)	−4.4 (2.7)	0.3 (1.0)	−0.2 (1.3)	*r* = 0.89, *P* < 0.0001	*r* = 0.83, *P* < 0.0001	*r* = −0.29, *P* = 0.0007
CORSI	Mean Location Error (z)	2.0 (1.7)	−0.2 (0.8)	0.3 (1.5)	*r* = −0.77, *P* < 0.0001	*r* = −0.64, *P* < 0.0001	*r* = 0.09, n.s.
DSST	Number of Correct Responses (z)	−2.3 (1.1)	−0.2 (0.9)	−0.0 (1.2)	*r* = 0.86, *P* < 0.0001	*r* = 0.87, *P* < 0.0001	*r* = 0.10, n.s.
TMT	Average Completion Time for TMT‐A (z)	4.3 (5.3)	0.5 (1.8)	0.2 (1.3)	*r* = −0.79, *P* < 0.0001	*r* = −0.82, *P* < 0.0001	*r* = −0.12, n.s.

*Notes*: All metrics were normalized by age‐matched normative data (i.e., participants who are EHC2). All metrics are reported in group mean and standard deviation. * Indicates *P* values below <0.001. The effect size for group comparison is the rank‐biserial correlation coefficient. See Table S1 in supporting information for the extended table, including all cognitive metrics and the rank‐biserial correlation coefficients for group comparison with EHC2.

Abbreviations: AD, Alzheimer's disease; CORSI, Freestyle Corsi tapping task; DSST, Digital Symbol Substitution Test; EHC, elderly healthy control; OCTAL, Oxford Cognitive Testing Portal; OIS, Object‐in‐Scene task; OMT, Oxford Memory Test; ROCF, Rey–Osterrieth Complex Figure; TMT, Trail Making Test.

### 2.3 Measurements of plasma biomarkers: Aβ42, Aβ40, p‐tau181, NfL, and GFAP

Four plasma biomarkers were assayed:

Amyloid pathology (“A”): Aβ42, Aβ40, and the Aβ42/40 ratio, which is a better measure of amyloid pathology than Aβ42 alone.^6^, ^37^

Tau pathology (“T”): p‐tau181, which is a specific and sensitive marker of tau pathology in the blood and is highly predictive of tau PET positivity.^11^

Neurodegeneration (“N”): NfL, the most commonly used blood‐based biomarker reflecting the rate of neurodegeneration occurring in the brain.^38^

Astrocytosis: GFAP, an established marker of astrocytosis and synaptic plasticity.^16^

#### 2.3.1 Plasma biomarker analysis

Blood was collected in six ethylenediaminetetraacetic acid (EDTA) tubes (10 mL each), and centrifuged (1800 g, room temperature, 10 minutes). The EDTA tubes were filled completely and gently inverted after collection to avoid coagulation. After centrifugation, plasma from all six tubes were transferred into one 50‐mL polypropylene tube, mixed, aliquoted into 0.5 mL polypropylene tubes (Fluid X, Tri‐coded Tube, Azenta Life Sciences), and stored at 4°C, until (< 8 hours) it was transferred into a −80°C freezer. The time between blood collection and centrifugation was <30 minutes. Transfer time between 4°C and −80°C storage was < 20 minutes, and the samples were kept refrigerated during transport. All cryovials were anonymized, and the unique cryovial code was logged into a secure database, linked to the participant's anonymous code and visit number.

Samples were shipped in dry ice to the Biomarker Factory/Fluid Biomarker Laboratory, UK Dementia Research Institute at University College London (UCL), London. The Dementia Research Institute (DRI) laboratory staff carried out the analyses. Plasma Aβ40, Aβ42, GFAP, and NfL were measured by single‐molecule array (Simoa) technology using the Neurology 4‐plexE assay on an HD‐X analyzer (Quanterix), according to manufacturer's instructions. Plasma p‐tau181 was also measured by Simoa using the pTau‐181 Advantage assay on an HD‐X analyzer (Quanterix). Further information regarding the analysis pipeline can be found in Methods S2 in supporting information.

### 2.4 Face‐to‐face neuropsychological screening

All participants completed the ACE in person at the time of the visit as a standard clinical screening test of cognition. ACE scores < 88/100 are considered abnormal, and all healthy controls scoring below that threshold were excluded from this study. However, patients with AD were not recruited based on a fixed threshold on standard cognitive testing but rather took part in the study according to the criteria outlined in paragraph 2.2, following clinical and radiological principles.

### 2.5 Digital cognitive test battery: Oxford cognition online platform

**FIGURE 2:**
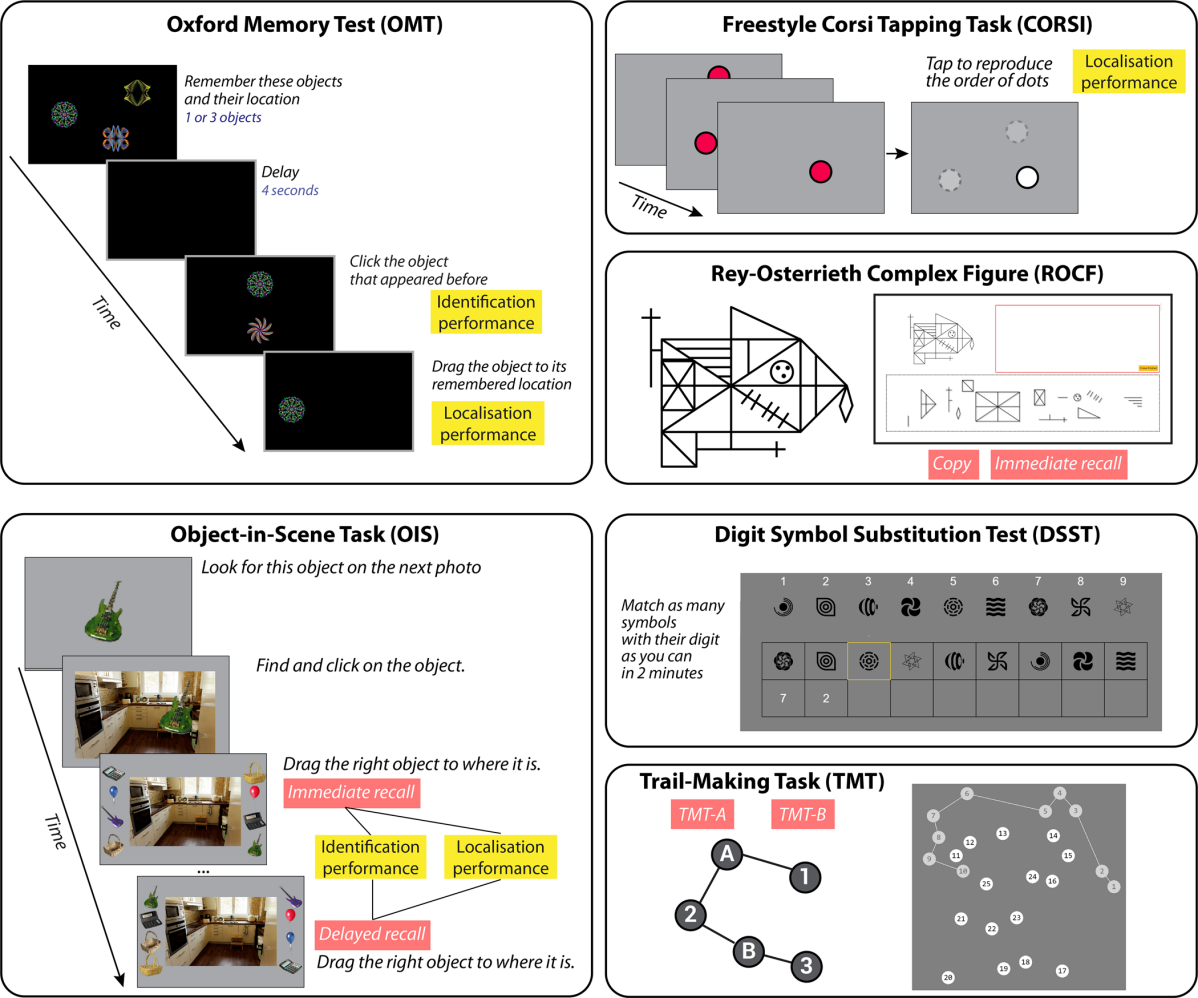
Experimental design of online digital cognitive tasks. Oxford Memory Test (OMT) is a “What was where?” visual short‐term memory experiment in which participants are presented with one or three fractal patterns positioned at various locations on‐screen for 3 seconds. After a 4‐second delay, participants identify the fractal pattern shown before and move it to its remembered location. Thus, the response reflects how precisely the memory was recalled. Additionally, the Object‐in‐Scene task (OIS) measures long‐term memory. Participants are shown a photo of an everyday scene and asked to remember a particular object placed in the picture. Another memory task is the Freestyle Corsi Tapping Task (CORSI), which involves remembering a sequence of random locations on the screen. Participants are then free to click anywhere on‐screen to indicate the remembered sequence of locations. Participants in the Rey–Osterrieth Complex Figure (ROCF) test their visuospatial abilities by dragging 13 elements given to copy a complex line graph on an empty canvas. Digit Symbol Substitution Test (DSST) requires participants to match symbols to digits according to a key located at the top of the page. More correct matches were made in under 2 minutes indicating faster processing speed. Trail Making Test (TMT) is also an executive function task. Participants are instructed to connect 25 circled numbers by clicking the circles in order as fast as possible. This task contains three trials of Task A (where the order is 1‐2‐3‐4‐5‐6‐…) and three trials of Task B (order 1‐A‐2‐B‐3‐C‐…). Full versions of all tasks can be tried online at https://octalportal.com.

Participants also completed a sequence of computerized cognitive tasks from OCTAL (Oxford Cognitive Testing Portal, available at https://octalportal.com; Figure 2). The tasks include the Oxford Memory Test (OMT), Object‐in‐Scene Memory Task (OIS), drag‐and‐drop version of Rey–Osterrieth Complex Figure (ROCF), Trail Making Test (TMT), Digit Symbol Substitution Test (DSST), and Freestyle Corsi Tapping Task (CORSI; Figure 2). They measure distinct aspects of human cognition, various forms of memory, attention, and executive functions. They were adapted from established behavioral paradigms or neuropsychological tests, while being robust against the type of device that a person is tested on. These six tasks can be tried at https://octalportal.com. The tasks were conceived and designed by S.Z. and M.H. Most of the tasks were built using the PsychoPy Builder (PsychoJS, version 2022.2.4) with custom‐written codes in Javascript, with one exception: the ROCF (see details below), which was fully custom written in HTML5 with JavaScript. All tasks were hosted on the server system Pavlovia.org.

A link with a unique patient and visit identifier was sent to the participants’ e‐mail address the same day as the in‐person visit when blood was collected. Participants were required to use Chrome or Firefox browsers on a desktop, laptop, or tablet with any operating system. They were encouraged to complete the online tests within a week maximum. After 2 weeks, the completion of the online tests was reviewed, and participants who did not complete the tasks within that time frame were prompted via e‐mail to do so. Tests completed > 3 weeks after the blood samples were discarded.

#### 2.5.1 Oxford Memory Task

OMT is the “What was where?” visual short‐term memory experiment, which has been described in previous publications.^4^, ^39^, ^40^ In this remote online version, participants were presented with one or three fractal patterns positioned at various locations on screen for 3 seconds (Figure 2). Then, a 4 second delay was accompanied by a black screen. Subsequently, one of these fractal patterns was shown alongside a foil pattern. The two patterns were shown along the vertical meridian with 4 cm separation, with the order of the target and foil randomized across trials. Participants must identify which pattern they just saw (identification performance) by clicking the target pattern and drag it to its proper location on the screen (localization performance). The foil was not a novel pattern; rather, it was part of the general pool of fractal images presented across the experiment. But the exact color and shape of the foil never showed up as one of the patterns to remember.

Each participant performed a practice block of six trials including three trials with one item followed by three trials with three items. This is followed by a main test block of 40 trials, including 20 trials of one item and 20 trials of three items. The order of trials was randomized online. No feedback was given during practice or main test blocks. Fractal stimuli were drawn from a library of 196 pictures of fractals (http://sprott.physics.wisc.edu/fractals.htm), including 49 different shapes and each shape contained four color variations.

As participants did the task remotely with their own devices, to ensure that the size of stimuli was physically the same across different devices, a card calibration procedure, previously described and validated,^41^ was used prior to certain tasks. Participants are instructed to place a bank card or card of comparable size on the screen, and adjust the slider until the size of the image of the card on the screen matches the size of the physical card. This allows us to estimate screen distance by calculating the display's logical pixel density in pixels per centimeter. After successful calibration, the diameter of the fractal stimulus is 2 cm. A MATLAB script (MathWorks, Inc.) was used to determine the fractals' locations in a pseudorandom manner with a few constraints. To avoid crowding and create a clear zone around the items' original locations, which is essential for the analysis of localization errors, fractals were never placed closer than 3 cm to one another.

Eight cognitive metrics were extracted from this task: identification accuracy (proportion of correct object identification, see Figure 2), location error (distance between response and target), identification time (reaction time to identify target), localization time (reaction time to place object), target detection (rate of detecting correct object and placed at target location), misbinding (rate of placing target at a non‐target location), guessing (rate of placing target randomly), and imprecision (how precise spatially is the response).

#### 2.5.2 Object‐in‐Scene Memory Task

This test provides measures of identification accuracy, precision of spatial localization, and semantic accuracy in visual short‐term and long‐term memory (Figure 2). Participants were presented with a photo of an everyday scene and instructed to remember a particular object placed in the picture. To aid effective encoding, the participant was also asked to click on the displayed object. Subsequently, 20 different objects were presented, and the participant was asked to choose the correct object and place it in the remembered location in the scene. To ensure that they were not simply remembering the name of the object, the object pool contained a foil that matched the target's category (e.g., two guitars of different color and shape). After five different object and scene pairs, participants were asked to reproduce the object–scene associations probed (delayed recall, after 3–4 minutes). There was a total of 20 trials divided into four blocks, and the order of the pairs was randomized.

Three metrics were extracted from the task for both immediate and delayed recall stages: object identification accuracy (proportion of trials in which participants correctly identified the original object; chance level = 5%), semantic identification accuracy (proportion of objects correctly recalled as belonging to the same semantic category as the target; chancel level = 10%), location error (the distance from the original target item location to the center of participant's response location; centimeter as unit).

#### 2.5.3 Rey–Osterrieth Complex Figure

This task is a digitized version of the traditional pen‐and‐paper test,^42^ which is an established measure of visuospatial abilities (Figure 2). The original ROCF task requires the participant to draw a complex line drawing freehand, first by replicating an existing figure (copy), and then again from memory (immediate recall). Our digitized version does not require hand drawing. Instead, the figure is split into 13 independent elements, and participants are required to drag each element into an empty canvas to copy the figure. Each test was automatically scored using an offline MATLAB‐based algorithm. In contrast to the discrete score used in the pen‐and‐paper version, the score of our digital version provides a continuous measure of precision. The middle large rectangle is selected as the anchor point as a reference element. If the element is not placed (not present on the canvas), there will be no score; otherwise, the distance from the large rectangle is computed. As a measure of imprecision for each element, the absolute difference between the ideal distance and the actual distance is then calculated. The absolute error is then scaled using a logarithmic function: if the element is placed relatively correctly, the difference in the distance from the big rectangle is computed; if the element is placed too far, the score is zero. The normalized absolute difference is then subtracted from 1 to calculate the score for this element. The sum of all element scores is 13, but the results are scaled to a percentage to match the original 36‐element picture. Our version's scoring is consistent with the pen‐and‐paper scoring guide, as the participant receives 1 point for correctly positioning the element and no score if the element is placed incorrectly or not at all on the canvas. This task and scoring have been validated with the in‐person traditional 36‐item pen‐and‐paper test and manual scoring with standard scoring guide in healthy participants before the start of the study. The percentages obtained at the copy and immediate recall—ROCF copy and recall scores—were used as metrics of interest.

#### 2.5.4 Digit Symbol Substitution Test

DSST provides a measure of processing speed. In this digitized version, participants were required to match symbols to digits according to a key located at the top of the page (Figure 2). The key consisted of nine symbols next to the digits 1 through 9. At the bottom of the screen, there was a row of nine randomized symbols. Participants reported the digit that corresponded to each symbol by clicking on the correct digit. The row was refreshed once all nine were answered. Participants were allowed 2 minutes to do as many matchings as they could and the number of correct matvhes within the allowed time (DSST‐correct responses) was used as variable of interest.

#### 2.5.5 Trail Making Test

TMT is a standard test of processing speed and executive functions.^43^, ^44^ In this online version (Figure 2), 25 circled numbers are presented on screen, and participants are instructed to connect them by clicking the circles in order as fast as possible. It contains three trials of Task A (where the order is 1‐2‐3‐4‐5‐6‐…) and three trials of Task B (order 1‐A‐2‐B‐3‐C‐…). Each participant sees six different trail maps randomly chosen from a pool of 100 pre‐made maps, generated using a “divide‐and‐combine” approach.^45^ The task also included a control condition of four trials to assess basic motor speed, in which participants are presented with two circles located at two opposite corners of the screen. One is labeled with 1 and the other with 2, and participants were instructed to connect 1 with 2 as quickly as possible. The average of the reaction times of the TMT was used as a variable of interest.

#### 2.5.6 Freestyle Corsi Tapping Task

This task is a modification of the Corsi Block Tapping Task,^46^ which is a standard measure of visual short‐term memory. In the original version, participants were presented with a set of nine identical wooden blocks positioned on a board. Subjects were required to point at the blocks in the order they were presented. They started with sequences of smaller blocks, and the number of blocks increased during the test. In the most common computerized version of the task, the participant is shown several identical blocks that are in fixed locations spread across the screen.^47^ Blocks then light up in sequence and the participant must remember which blocks lit up and in what order. In this digital version, the blocks’ locations were not fixed (“Freestyle Corsi Tapping Task”). In an *n*‐location trial, a 1 cm–wide red dot appeared at a random location on the screen, disappeared after 1 second, and reappeared at another random location on the screen (for *n* > 1), and this process was repeated *n* times up to a sequence of five items. Once the sequence has finished, after a 1 second break the participant could freely click anywhere on screen to indicate where each dot appeared in sequence. The location error was calculated as the average distance between the response and the target location. The task was divided into five blocks, each block having five trials of an *n*‐location sequence (i.e., five blocks of one item, five blocks of two items, up to five blocks of five items). The average of the reaction times of the five conditions was chosen as a variable of interest.

### 2.6 Questionnaire‐derived motivation and mood metrics

All participants also completed two questionnaires which were hosted on Qualtrics: (1) Apathy Motivation Index (AMI), an 18‐item questionnaire, subdivided into three subscales of apathy: emotional, behavioral, and social apathy^48^ and (2) Geriatric Depression Scale (GDS), short form, which includes 15 questions. It is a screening tool designed to assess depressive symptoms in elderly people. A total score > 5 indicates probable depression.^49^

### 2.7 Attention checks

Attention checks were incorporated throughout the study: (1) In OIS, participants were required to adhere to the instruction of dragging an object onto the scene immediately after viewing the object and the photo. A low‐effort response was defined as object identification accuracy < 20% (10% was the chance level for the correct object and 20% was the chance level for the correct semantic category). No participant failed this check. (2) In OMT, a failure of attention check was defined as an unreasonably short localization time (cut‐off at 0.2 second). Eight EHC2 participants failed this check. (3) In DSST, if the correct rate was < 20%, it meant the participants did not follow the instruction to match the digit with the symbol based on the key provided (the chance level for pure guessing would be 11%). Four EHC2 participants failed this attention check. (4) In DSST, if the participant stayed idle for more than 60 seconds in total, it was also considered a failure of attention check. One of the four participants who failed the last attention check also failed this check. (5) Healthy controls should be able to achieve a full score on the ROCF copy. If a healthy control scored < 50%, their copy and recall would be excluded. All participants passed this check. (6) Each of the two questionnaires had a validation question “This is a validation question. Please choose ‘Completely untrue.’” No one failed this attention check. While no participant failed all six attention checks, four individuals were excluded after failing more than three of them. The performance of the tasks on which attention checks failed was discarded.

### 2.8 Statistical analysis

For analysis and data visualization purposes MATLAB (version R2023a), R studio (version 12.0), JASP (version 0.16.4), and SPSS (version 29.0) were used. Demographics, cognitive tests, and plasma biomarker levels were compared using a Mann–Whitney *U* test for continuous variables, while χ^2^ test was used for categorical variables such as sex. *P* values were two‐tailed with statistical significance set at *P* < 0.05 for all analyses. Rank‐biserial correlation was used for effect size estimation. If data from multiple visits were available, averaged values per participant across visits were used.

#### 2.8.1 Age‐adjusted digital cognitive measures

*Z* score (i.e., number of standard deviations from the mean of the normative population in the similar age [±3 years]) was computed for each variable and each subject, based on a normative population of 352 online participants > 50 years old (EHC2, see Table 1 for demographics). On average, each individual's performance was adjusted with 55.8 (standard deviation [SD] 14.7, min 30, max 81) participants from the EHC2 group.

#### 2.8.2 Correlation between digital cognitive metrics and plasma biomarkers

Plasma biomarkers’ log10 transformed values and *Z* scores of digital cognitive measures were used for correlation and linear regression analyses. Correlations between digital cognitive metrics and plasma biomarkers were assessed with Spearman rank test, using age, sex, and education as covariates. The Benjamini–Hochberg method, which controls the false discovery rate (FDR), was used to correct for multiple comparisons.

#### 2.8.3 Machine learning for group classification

Additionally, machine learning was applied to predict group classification and plasma biomarkers levels, first using MATLAB‐based algorithms for feature ranking, to estimate the absolute contribution of each variable. The fscchi2 function in MATLAB (univariate feature ranking for classification using chi‐square tests) was used to predict group classification, while the fsrftest function (univariate feature ranking for regression using *F* tests) was used to predict continuous variables, that is, plasma biomarker levels. Rank importance scores were then transformed into *P* values by calculating the exponential of the negative scores. Second, we applied the R‐based MuMIn package to test which combinations of biomarkers would best predict group classification and plasma biomarker levels. For predicting groups, we used logistic regression, while for predicting p‐tau181 level and Aβ42/40 ratio linear regression was deployed. The MuMIn package then uses the dredge function to achieve model selection, with the best‐performing model having the lowest corrected Akaike information criterion (AIC). Receiver operating characteristic (ROC) curves and areas under the ROC (AUCs) were then computed for the model of interest. The pROC package in R with De Long test was used to compare model performance in direct comparisons between two ROC curves.

## 3 RESULTS

### 3.1 Participants’ test and plasma biomarker overview

**FIGURE 3:**
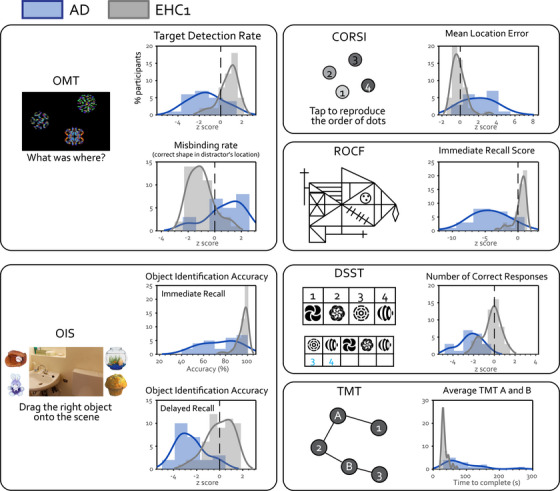
Digital cognitive metrics. In all online tasks, patients with AD (plotted in blue) performed significantly worse and had higher variability compared to age‐matched healthy controls (EHC1, plotted in gray). On the X axis, cognitive performance is shown as *z* scores derived from age‐matched online normative data, except TMT for which the raw completion time is shown. Y axis indicates the percentage of participants. See supporting information for the distribution plots for all other cognitive metrics. AD, Alzheimer's disease; CORSI, Freestyle Corsi tapping task; DSST, Digit Symbol Substitution Test; EHC, elderly healthy control; OIS, Object‐in‐Scene; OMT, Oxford Memory Test; ROCF, Rey–Osterrieth Complex Figure; TMT, Trail Making Test.

Patients with AD performed significantly worse in all digital cognitive metrics with high effect sizes (rank‐biserial correlation, or rrb > 0.5) compared to matched controls (EHC1); see Figure 3 for distribution comparison for key cognitive metrics, and Figures [Link], [Link] in supporting information for all metrics and online normative data. Patients with AD showed a large deficit in executive functions, indexed by TMT and DSST, as indicated by an average 8.5 SD below expectation. They also were 2.0 to 7.5 SDs below expectation in both identification and localization of recalling remembered items in short‐term memory (OMT and CORSI) and long‐term memory (OIS delayed recall). Noticeably, patients with AD were particularly impaired at memory recall (OIS and ROCF‐recall). For example, both EHC groups could normally recall >90% of objects correctly with a very precise spatial memory (1 cm location error); in contrast, although AD remembered the object's semantic category (e.g., it was a guitar), they could only recall 72.9% of the objects (but which guitar?) accurately with an average location error of 7.5 cm away from the center of the object (which is 2 cm wide). Similarly, EHCs recalled 80% of ROCF at immediate recall, but patients with AD on average scored < 50% (5.2 SD below expectation).

Compared to the online participants (EHC2), EHC1 performed slightly but significantly better in many cognitive metrics (see Table S1). In our sample, this difference could not be explained by age, education level, or the testing environment (all completed remotely at home anonymously). EHC2 performed particularly worse in the OMT, on which they made significantly more misbinding errors and were faster at localization compared to the participants we tested locally. This group difference might be due to a speed–accuracy trade‐off in the EHC2 group; in this online group, participants with shorter localization time made associated with more misbinding errors (Pearson *r* = −0.22, *P* = 0.003), while in contrast no correlation between speed and accuracy was found in EHC1 (*r* = −0.07, *P* = 0.64).

Regarding plasma biomarkers, as expected, patients with AD had higher mean levels of p‐tau181, GFAP, and NfL, and lower Aβ42/40 ratio compared to cognitively unimpaired controls (Table 1).

ACE scores were significantly different between AD and EHC1, and had a high effect size in group comparisons, which was expected as it was the only test used for diagnosis (Table 1). Patients with AD were in general more apathetic and depressed compared to EHCs (Table 1).

### 3.2 Relationships between plasma biomarkers and cognitive metrics

**FIGURE 4:**
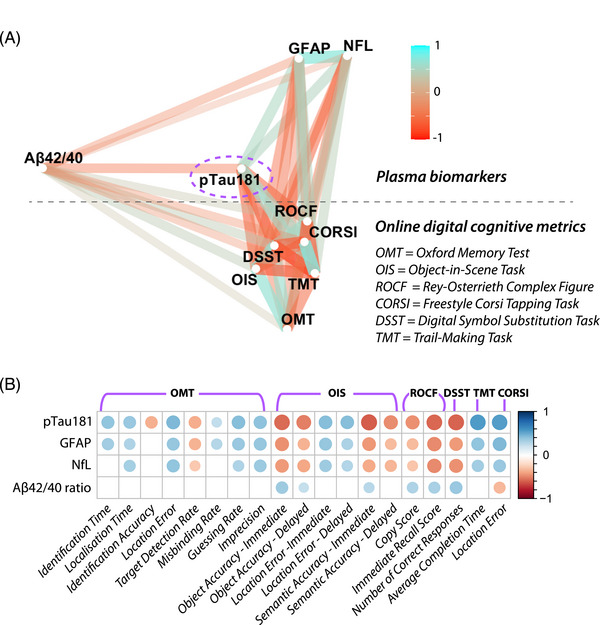
p‐tau181 shows the strongest relationship with digital cognitive tests. A, Network plot of relationships between all plasma biomarkers (clustered on the top left) and online digital cognitive tasks (clustered on the bottom right). Associations are presented in graded colors, where red is associated with a negative correlation and light blue with a positive correlation. The shorter distance between two metrics indicates a stronger relationship (larger correlation coefficient). For online tasks, only one metric was selected per task, according to the highest effect size in discriminating between groups. B, The strength of the correlation is given by the diameter of the circle, with positive correlations in blue and negative in red. All displayed correlations are significant after Benjamini–Hochberg correction for multiple comparisons. Aβ, amyloid beta; AD, Alzheimer's disease; GFAP, glial fibrillary acidic protein; NfL, neurofilament light chain; p‐tau, phosphorylated tau.

The relationships between all plasma biomarkers and digital cognitive metrics are visualized as a network plot in Figure 4A, in which the strength of the relationship is represented by the distance between the metrics. Among the four plasma biomarkers investigated in the present study, p‐tau181 was most strongly correlated with our digital cognitive metrics, which all clustered on the bottom right of the plot. In contrast, the Aβ42/40 ratio showed the weakest relationship with cognitive performance as well as with the other three plasma biomarkers.

This pattern of relationships can also be appreciated when looking at the individual correlation between each pair of biomarkers and cognitive metrics (Figure 4B). Across the different tasks examined, multiple metrics of short‐term memory were correlated with p‐tau181, GFAP, and NfL levels; the better the performance, the lower the levels of these three plasma biomarkers. Similarly, these plasma levels were also correlated with executive function metrics such as DSST and TMT, and with visuospatial ability as indicated by the ROCF copy score. In contrast, Aβ42/40 ratio was only weakly associated with selected short‐term memory metrics and long‐term memory metrics in OIS (e.g., immediate and delayed recall accuracy, immediate semantic accuracy) and performance at the Freestyle Corsi Tapping Task. Aβ42/40 ratio levels also correlated with visuospatial abilities (copy and immediate recall scores of ROCF) and processing speed (DSST). These results survived multiple comparison corrections across 76 correlations. Within‐group correlations between cognitive tests and plasma biomarkers did not survive corrections for multiple comparisons. Within‐group correlations for EHC1 and AD, uncorrected for multiple comparisons, are shown in Figure S3 in supporting information.

### 3.3 Which plasma/cognitive metric best predicts AD?

**FIGURE 5:**
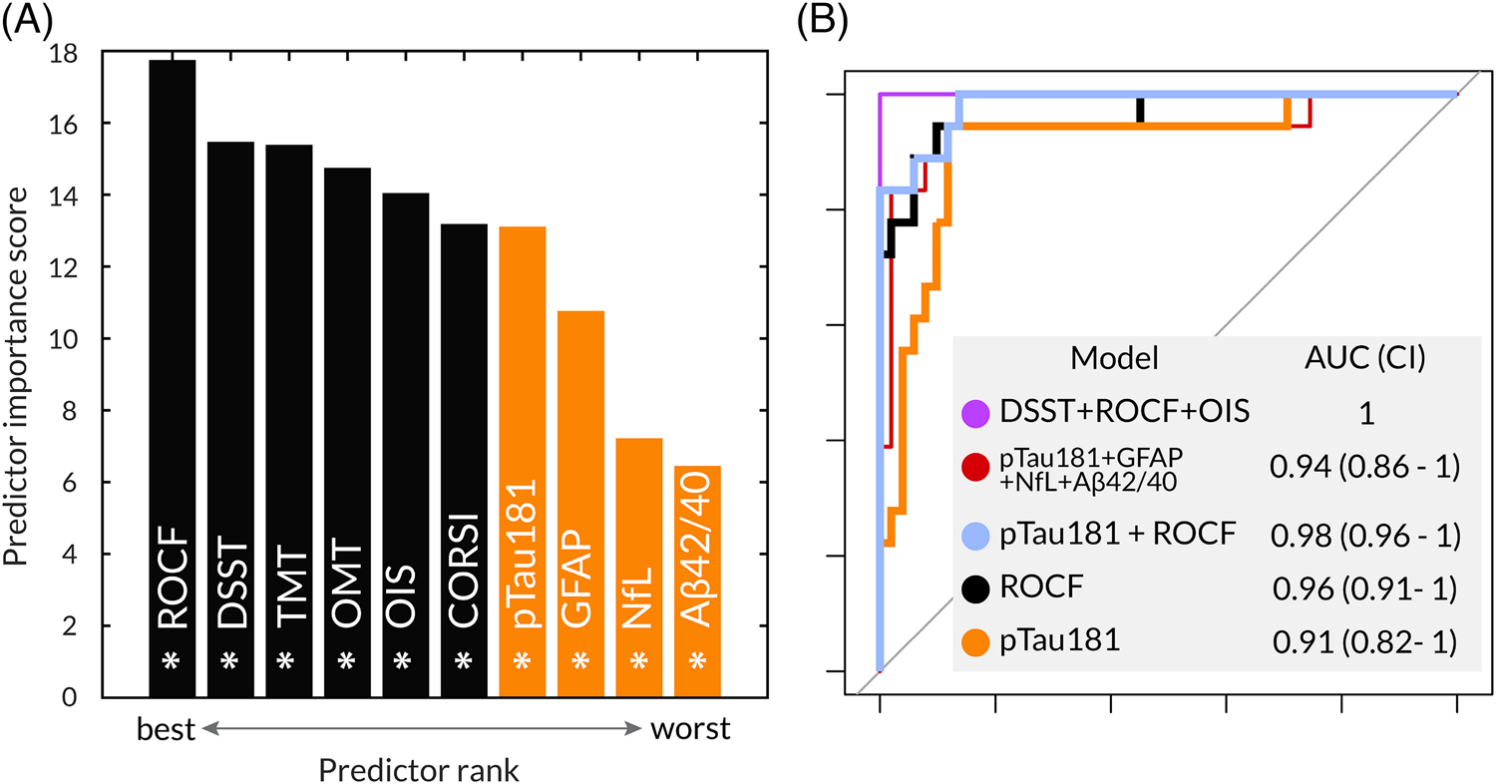
Which cognitive metric or plasma biomarker best predicts AD? A, Ranked biomarkers and digital cognitive metrics in predicting group AD or control. Plasma biomarkers are marked in orange, while online cognitive metrics are marked in black. All metrics were significant predictors of group classification. OMT = Identification accuracy of the Oxford Memory Task, OIS = Object Identification Accuracy in Immediate Recall of the Object‐in‐Scene Memory Task, ROCF = recall of the Rey–Osterrieth Complex Figure, DSST = number of correct responses of the Digit Symbol Substitution Task, TMT = average reaction time of the Trail Making Test, CORSI = average location error of the Freestyle Corsi Tapping Task. B, ROC curves for group classification. Light blue shows the combined model with ROCF and p‐tau 181, the black line indicates ROCF alone model and orange shows the model with p‐tau181 alone. The winning model, with DSST, ROCF and OIS, is displayed in a thinner, purple line. The model with the combination of all plasma biomarkers is depicted in a thinner, red line. Aβ, amyloid beta; AD, Alzheimer's disease; AUC, area under the curve; CI, confidence interval; GFAP, glial fibrillary acidic protein; NfL, neurofilament light chain; p‐tau, phosphorylated tau.

The selected variables were then ranked according to their importance in predicting group classification, that is, AD or EHC1 (Figure 5A) using chi‐square tests. The rank represents the negative log of the *P* values. In this sample, all cognitive metrics ranked higher than plasma biomarkers. All tests and biomarkers were significant predictors of the group (all *P* < 0.001 except Aβ42/40 ratio, *P* = 0.002).

We then explored which were the best predictors of tau and amyloid pathology, indexed respectively by p‐tau181 and the Aβ42/40 ratio. Most of our digital cognitive tests, except OMT, were better predictors of p‐tau181 levels compared to other plasma biomarkers such as GFAP and the Aβ42/40 ratio (Figure S4A in supporting information). The best‐performing test in predicting p‐tau181 levels was DSST, followed by OIS, TMT, and ROCF (all *P* < 0.001). All plasma biomarkers were significant predictors of p‐tau181 levels; NFL (*P* < 0.001), GFAP (*P* < 0.001), Aβ42/40 (*P* = 0.002).

Conversely, p‐tau181 was the only statistically significant predictor of amyloid burden (*P* = 0.018; Figure S4B). The best performing digital cognitive test to predict amyloid burden was DSST, but it was not statistically significant (*P* = 0.092).

Model comparison using the MuMIn R function was then used to choose the best combination of plasma biomarkers and cognitive metrics in predicting group classification (using logistic regression), p‐tau181, and the Aβ42/40 ratio levels (linear regression). The best model for predicting groups (the one with the lowest [AIC]), consisted of recall of the ROCF, Object Identification Accuracy – Immediate recall of the OIS, and DSST (Figures 5B and S4C), with an AUC of 1. When Lasso penalization was introduced to avoid perfect separation of the best model, it still performed significantly well (AUC = 0.93).

If taken individually, the recall of the ROCF had an AUC of 0.962, DSST had an AUC of 0.955, and Object Identification Accuracy – Immediate recall Accuracy of the OIS had an AUC of 0.937, while p‐tau181 had an AUC of 0.911 in predicting group classification. While ROCF alone was not statistically significantly different from the best model (ROCF: *Z* = 1.43, *P* = 0.15), the model which incorporated DSST, ROCF, and OIS was better than a model containing only p‐tau181: *Z* = 2.0, *P* = 0.04). However, the model containing only ROCF was not better compared to the model with p‐tau181 alone (*Z* = 1.00, *P* = 0.31). If p‐tau181 and ROCF were combined, this combination achieved an AUC of 0.983, and there was a non‐significant trend toward the combination being statistically superior to p‐tau181 alone (*Z* = −1.86, *P* = 0.06). If all digital cognitive tests were combined the model achieved an AUC of 1, while if all plasma biomarkers were combined that resulted in an AUC of 0.940. These were, however, not statistically different (*Z* = −1.41, *P* value = 0.16).

In comparison, ACE had an AUC of 0.97 in discriminating between groups, which was, however, not different compared to the best performing digital metric, ROCF (*Z* = 0.99, *P* value = 0.82), nor to the best model (*Z* = −0.23, *P* value = 0.32).

The best model for predicting p‐tau181 levels consisted of Aβ42/40, DSST, OIS, ROCF, and OMT, which had an adjusted *R* ^2^ of 0.50 (Figure S4C). If single metrics were evaluated, in predicting p‐tau181 levels, ROCF, OIS, and DSST had an adjusted *R* ^2^ of, respectively, 0.31, 0.34, and 0.38, while in comparison, ACE had an adjusted *R* ^2^ of 0.24. AICs were −7.55 (ROCF), −10.78 (OIS), −15.22 (DSST), and −1.2 (ACE), with the best‐performing model being the one containing DSST (more negative). The winning model for predicting Aβ42/40 levels consisted only of p‐tau181, but had an overall poor model fit with an adjusted *R* ^2^ of 0.14 (Figure S4C).

## 4 DISCUSSION

Currently, there are no published studies, to the best of our knowledge, which have reported on the relationship between performance on a panel of online cognitive tests and plasma biomarkers of AD. The findings reported here demonstrate that patients with AD have impaired performance on our digital cognitive tests and that this is related to pathological blood‐based biomarkers of the disease (Figures 3 and 4). Levels of plasma p‐tau181, GFAP, and NfL were all highly correlated with several cognitive metrics, with p‐tau181 being the biomarker that showed the closest association to digital cognitive performance (Figure 4A). Overall, the results of this study show that digital testing is a promising avenue to measure cognitive functions in AD, which would have the potential to make a significant impact on several fronts. Screening for the disease, recruitment and stratification into clinical trials, and longitudinal follow‐up in intervention studies could all be transformed if cognitive testing were to be conducted robustly, remotely, and frequently.^5^ Digital cognitive testing could make this happen.

Digital platforms are emerging as potential screening and diagnostic tools for people at risk of developing AD.^50^ Most studies using such platforms have focused on screening healthy individuals.^51^ Moreover, biomarker validation on these digital platforms is mostly limited to one single marker, frequently amyloid PET.^51^ Some brief digital screening tools have demonstrated promise in differentiating amyloid‐positive and tau‐positive MCI patients (as measured by amyloid and tau PET) from MCI without evidence of amyloid or tau accumulation, but they have not been very good at separating healthy controls from people with MCI or prodromal AD.^52^ A shorter digital version of the Face Name Associative Memory Exam (FNAME) or FACEmemory^®^, measuring episodic memory, has been found to correlate with CSF levels of p‐tau181 and the Aβ42/40 ratio.^53^ Reduced learning over time over multiple exposures of the pen‐and‐paper FNAME has also been associated with amyloid and tau burden at PET^54^ and the Spanish version of the same test (S‐FNAME) has been shown not to be significantly correlated with plasma Aβ42/40 levels in a cohort of cognitively unimpaired individuals and patients with SCI, while its composite score (SNF‐F) showed a mild correlation (rho = 0.193, *P* = 0.006) in the same study.^55^ However, the performance of the online shorter version of these tests compared to plasma biomarkers of AD is currently unknown.

One of the strengths of the current study is the inclusion of tests measuring different cognitive domains and the use of different plasma biomarkers, measuring not only amyloid and tau accumulation but also neuroinflammation and neurodegeneration. To our knowledge, no published study has previously investigated the relationship between these four biomarkers and performance on a fully online platform in a mixed population of elderly healthy controls and patients with AD.

A key finding of this study is that digital cognitive metrics were more tightly correlated with p‐tau181 than the Aβ42/40 ratio (Figures 4 and S4). This is not entirely surprising, as amyloid burden has been shown to have a weaker association with cognition compared to tau.^56^, ^57^ The biological underpinning of such dissociation between small‐magnitude correlations between amyloid plaques’ deposition and cognition in contrast to the strong association with tau burden might be explained by the tighter association between tau accumulation, neuronal loss, and subsequent gray matter atrophy,^58^ as opposed to a relatively slow, indolent accumulation of amyloid burden over time.^59^

One possible explanation for the better performance of p‐tau181 compared to the Aβ42/40 ratio lies in the fact that p‐tau181 in blood correlates well with both amyloid and tau PET,^10^ and not only with amyloid burden. Moreover, unlike GFAP and NfL, p‐tau181 is AD specific.^11^ While tau accumulation in the medial temporal lobe has often been found to be associated with the decline of episodic memory performance at standard pen‐and‐paper tests,^58^ it is also increasingly recognized that a decline in visual short‐term memory performance at digital tasks, including one used in our battery, OMT, can be associated with medial temporal lobe atrophy in patients with AD.^4^ Our results extend previous findings to biological hallmarks of AD measured by plasma biomarkers, and to other novel visual short‐term memory tests, which perform better at a head‐to‐head comparison with the abovementioned test, OMT (Figure 5A).

A longitudinal increase in GFAP levels has been found to be associated with decline in memory, attention, and executive domains in cognitively unimpaired individuals at face‐to‐face tests, while in the same study an increase in NfL did not show such an association.^60^ Our results show that these digital metrics, measuring visual short‐term memory, long‐term memory, visuospatial copying, executive function, and processing speed, are associated with both GFAP and NfL cross‐sectional levels in a cohort of AD and HC, even if such associations were of lower magnitude compared to p‐tau181 (Figure 4A,B). Longitudinal studies are needed to see whether GFAP levels could be more tightly correlated compared to NfL to a decline of performance at these digital tasks in cognitively unimpaired controls.

Taking a closer look at single cognitive domains, a large meta‐analysis that investigated different indices of amyloid positivity in cognitively unimpaired elderly adults without blood‐based biomarkers showed that although episodic memory might be correlated with amyloid burden, global cognition and executive functions are not if assessed by amyloid PET.^61^ On our digital platform, higher amyloid burden indexed by a lower Aβ42/40 ratio was not uniquely associated with long‐term memory abilities but was correlated with performance across multiple tasks, measuring visual short‐term and long‐term memory, processing speed, and visuospatial function (Figure 4B). These results are encouraging, as they might suggest that these digital metrics are sensitive tools, showing a higher range of association with amyloid burden beyond episodic memory. However, similarly to pen‐and‐paper tests, these associations were of small magnitude and no associations were found in the sample of cognitively unimpaired individuals in this study after correcting for multiple comparisons across 76 correlations.

Crucially, in this sample all digital metrics were better predictors of group classification (AD or EHC1) compared to plasma biomarkers (Figure 5A). In determining group classification using logistic regression, three digital tests performed best, as shown by the best model (lowest AIC) automatically selected (via the MumIn package) using model comparison of all possible combinations of digital and plasma biomarkers (see Figure 5 and S4C). These were the recall on the ROCF test (a measure of visual episodic memory), Object Identification Accuracy – Immediate recall on the OIS task (a measure of visual short‐term memory), and DSST (a measure or processing speed of visual short‐term memory). If these three tests were used in combination, this best‐performing model had AUC of 1, achieving perfect separation of patients from controls (Figure 5B), which was better than the model containing p‐tau181 alone. However, the best‐performing digital metric, recall of ROCF, if used in isolation performed equally well as p‐tau181 in group classification. Of course, it is not surprising in this case that three cognitive tests together perform so well in distinguishing AD from healthy controls because of the separation in cognitive performance between these groups, as would be expected to be the case with any comparison between a group with established neurodegeneration and healthy people. A biological confirmation is needed to correctly identify patients with AD, and our cognitive metrics should not be considered a substitute for biological characterization. However, it is encouraging to observe that the combination of all our digital metrics performed equally as well as the combination of four different plasma biomarkers, and that the winning model for group classification contained three digital metrics and no plasma biomarker. Regarding prediction of p‐tau181 levels, DSST was the digital test which achieved the highest performance. We consider this result to be valuable as this test only takes 2 minutes to perform and might suggest that lengthy cognitive tests might not be necessarily needed to capture subtle biological variations.

Importantly, among the digital metrics, ROCF had comparable performance to the standard cognitive scores used (ACE) in group classification, and ROCF, OIS, and DSST had better performance (higher adjusted *R* ^2^ and lower AIC) than ACE in predicting p‐tau181 levels. This is encouraging, as in the future, the combination of these measures might be used as a proxy for standard cognitive metrics while saving a considerable amount of time in face‐to‐face appointments.

### 4.1 Limitations and future directions

There are also several limitations to this study. One is that most of EHC1 and patients with AD came from a White Caucasian background, are highly educated, and based in south‐east England. We tried to address this by enrolling a second independent dataset of EHC2 to gather additional information regarding online performance in a more representative sample. However, our findings should be replicated in other populations with greater ethnic and socioeconomic diversity and wider geographical spread.

The very high AUCs of these metrics in predicting groups in this small, highly selected sample might partially explain the lack of positive contribution of adding cognition to plasma biomarkers. With accuracy being at ceiling, further evidence is needed to establish whether combining p‐tau181 to digital metrics might be beneficial in a larger dataset including different populations such as individuals with SCI or MCI. Another limitation of the current study is that within‐group correlations between cognitive tests and plasma biomarkers did not survive correction for multiple comparisons, which might be potentially due to the small sample size. A bigger sample would also be required to assess the performance of these metrics in people with preclinical AD versus amyloid and tau‐negative cognitively unimpaired healthy controls. Therefore, whether the combination of digital cognitive metrics and plasma biomarkers can be useful to stratify which individuals in the preclinical or prodromal phase of AD might be at risk of developing AD dementia remains to be established. However, it is encouraging that, even with a relatively small sample size, these metrics show a good correlation with several plasma biomarkers, surviving multiple comparisons and corrections for age, sex, and education, which are major confounders in both plasma biomarkers and cognitive assessments.^62^, ^63^

The patient population included in this study was already largely at the AD dementia stage, when cognitive impairment is overt. In this sample, plasma p‐tau181 was the biomarker that was more closely associated with cognitive metrics and the best predictor of group classification. However, we cannot exclude that other biomarkers such as p‐tau217 could show an even higher association with cross‐sectional or longitudinal cognitive function in the same population, as some evidence suggests.^64^, ^65^ Also, early markers of amyloid deposition such as p‐tau217, p‐tau213, and GFAP may be more closely linked with cognitive changes in the early phases of the disease.^14^, ^15^, ^64^, ^65^

Finally, a limitation of remote testing is the lower level of control over experimental conditions at home compared to testing in hospital settings. In this study, patients with AD had different levels of cognitive impairment, and some patients needed external support to ensure they understood task instructions and could carry out the task. This support was delivered by the caregiver if tests were performed at home, or by a member of the team if the tests were done at the hospital. However, these are intrinsic limitations of remote testing and are not unique to our study.

## 5 CONCLUSION

To conclude, digital cognitive metrics were associated with several plasma biomarkers, particularly p‐tau181, but also with GFAP and NfL, and to a much lesser extent with the Aβ42/40 ratio. Adding these metrics to p‐tau181 did not improve group classification in this sample, but the best performing metric, the recall of ROCF, performed at par with p‐tau181 levels. As plasma biomarkers are being proposed as equivalent to CSF biomarkers in the forthcoming National Institute on Aging–Alzheimer's Association revised criteria for AD,^66^ and given their increased use in clinical practice,^67^ implementation of a digital cognitive platform that has been validated with AD plasma biomarkers provides an important step forward for future large‐scale deployment.

## AUTHOR CONTRIBUTIONS

**Sofia Toniolo**: conceptualization; participants’ recruitment; project administration; resources management; data collection and curation; analysis; data visualization; writing. **Sijia Zhao**: conceptualization; software development; code development; digital platform data curation and management; analysis; data visualization; writing. **Anna Scholcz**: cognitive testing data collection and curation. **Benazir Amein**: biomarker samples preprocessing and data curation. **Akke Ganse‐Dumrath**: cognitive testing data collection and curation. **Amanda J. Heslegrave**: biomarker samples analysis. **Sian Thompson and Sanjay Manohar**: participants’ recruitment. **Henrik Zetterberg**: biomarker samples analysis. **Masud Husain**: conceptualization; supervision; resources management; funding acquisition; participants’ recruitment; writing. All authors read and approved the final manuscript.

## CONFLICT OF INTEREST STATEMENT

Henrik Zetterberg has served on scientific advisory boards and/or as a consultant for Abbvie, Acumen, Alector, Alzinova, ALZPath, Annexon, Apellis, Artery Therapeutics, AZTherapies, Cognito Therapeutics, CogRx, Denali, Eisai, Nervgen, Novo Nordisk, Optoceutics, Passage Bio, Pinteon Therapeutics, Prothena, Red Abbey Labs, reMYND, Roche, Samumed, Siemens Healthineers, Triplet Therapeutics, and Wave; has given lectures in symposia sponsored by Cellectricon, Fujirebio, Alzecure, Biogen, and Roche; and is a co‐founder of Brain Biomarker Solutions in Gothenburg AB (BBS), which is a part of the GU Ventures Incubator Program (outside submitted work). The other authors declare no financial or non‐financial competing interests. Author disclosures are available in the supporting information.

## CONSENT STATEMENT

The study was performed in accordance with the ethical standards as laid down in the 1964 Declaration of Helsinki and its later amendments. Ethical approval was granted by the University of Oxford ethics committee (IRAS ID: 248379, Ethics Approval Reference: 18/SC/0448). All participants gave written informed consent prior to the start of the study.

## Supporting information

ICMJE Disclosure Form

Supporting Information

## Data Availability

De‐identified data supporting this study may be shared based on reasonable written requests to the corresponding author. Access to de‐identified data will require a Data Access Agreement and IRB clearance, which will be considered by the institutions that provided the data for this research. The source code will be shared using a Creative Commons NC‐ND 4.0 international licence upon reasonable written request to the corresponding author and requires a research use agreement. For the purpose of Open Access, the author has applied a CC BY public copyright licence to any Author Accepted Manuscript version arising from this submission.
