# Supplementary material for: Relationship of plasma biomarkers to digital cognitive tests in Alzheimer's disease
Source: Alzheimers Dement (Amst). 2024 Apr 14;16(2):e12590. doi: 10.1002/dad2.12590 (PMC11016819; doi:10.1002/dad2.12590)
Supplement: Supplementary file 2 — Supporting Information [file DAD2-16-e12590-s001.pdf]

Supplementary Materials

Supplementary Methods 1 | Socioeconomic status and ethnic group of EHC2 group 1
Table 1. Ethnic groups. 1
Figure 1. Distribution of subjective socioeconomic status in EHC2. 2
Supplementary Table 1 | Digital cognitive metrics 3
Supplementary Methods 2 | Processing pipeline for plasma biomarkers 5
Supplementary Figure 1 | Group comparison for all digital cognitive metrics in Oxford Memory Task (OMT) and Object-in-Scene Task (OIS)............7
Supplementary Figure 2 | Group comparison for all digital cognitive metrics in ROCF, DSST and TMT............8
Supplementary Figure 3 | Apathy and depression are not strongly correlated with plasma biomarkers or performance at digital cognitive tests............9
Supplementary Figure 4 | Which cognitive metric or plasma biomarker best predicts pTau181 or Aβ42/40 levels? 10

Supplementary Methods 1 | Socioeconomic status and ethnic group of EHC2 group

340 of the 352 EHC2 participants provided us with their ethnic group. The distribution of the EHC2 is closely aligned to the UK population, with a good coverage of four major ethnic groups [1].

Table 1. Ethnic groups.

"White" group includes Caucasian, Sephardic Jew, Mexican, Middle Eastern and Latino/Hispanic. "Asian" group include South Asian, East Asian and South East Asian. "Black" group include African Black and Caribbean Black. "Other" group include Mixed and other groups that were not listed above.

Ethnicity	AD	EHC1	EHC2
White	77.8%	66.0%	74.4%
Asian	2.8%	0.0%	7.9%
Black	0.0%	0.0%	10.3%
Other	19.4%	34.0%	7.4%
Ethnicity unknown	2.8%	3.8%	3.5%

Subjective socioeconomic status, as measured by the MacArthur Scale of Subjective Social Status, is a typical technique to get an understanding of the participants' socioeconomic position without directly measuring the objective socioeconomic status (e.g., household income, property ownership, etc.). It depicts a ten-rung ladder, with participants asked to place

themselves on one of the rungs by determining where they stand in relation to other people in their country [2] and has shown good test-retest reliability and is expected to be strongly associated with objective socioeconomic status [3]. The distribution of EHC2's subjective socioeconomic status is depicted in Figure 1 below.

Figure 1. Distribution of subjective socioeconomic status in EHC2.

204 of 352 participants provided their answer to the MacArthur Scale of Subjective Social Status. The group mean was 5.7 (1.5) which was within the expected range of the UK population.

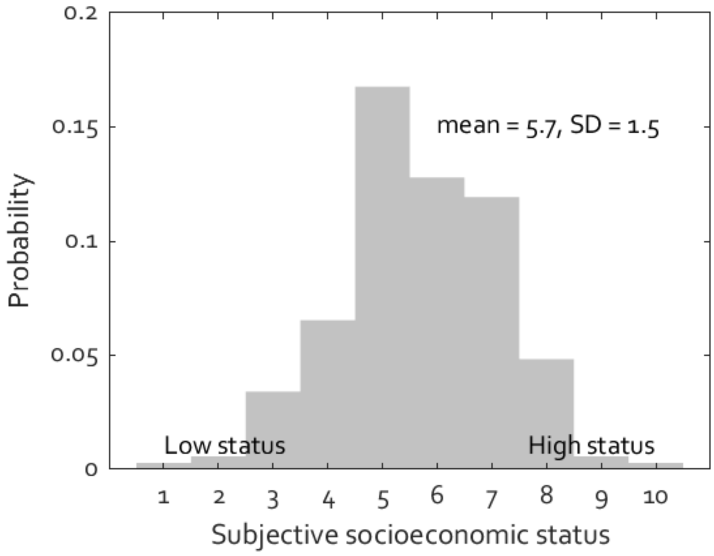

Even though an up-to-date distribution of subjective socioeconomic status in the UK is not publicly available, one way to estimate the average norm of subjective socioeconomic status is based on the countries with gross domestic product (GDP) per capita close to the UK. According to a recent social science report containing over 33,000 responses from 29 countries worldwide, GDP per capita is a strong predictor of average subjective socioeconomic status [4]. The GDP per capita of Sweden, the Netherlands, and Finland was very similar to that of the UK, and their average normative subjective socioeconomic status ranged between 5.6 and 6.0. The new EHC2 group's average socioeconomic level is 5.7 (SD=1.5), which falls within this expected range for the UK population. This, although indirect, implies that, in terms of socioeconomic position and ethnic groups, our EHC2 is a good general population representative.

Supplementary Table 1 | Digital cognitive metrics

The raw measures are followed by normalised measures.

Digital Cognitive Tasks	Metrics	AD (n= 46)	EHC 1 (n= 53)	EHC 2 (n=352)	AD vs EHC 1	AD vs EHC 2	EHC 1 vs EHC 2
OMT	Identification Accuracy (%)	81.3 (10.1)	92.3 (4.9)	89.6 (7.7)	rrb = 0.63, p <0.0001	rrb = 0.47, p = 0.0001	rrb = -0.21, n.s.
	Identification Accuracy (z)	-1.3 (1.9)	0.6 (1.0)	-0.2 (1.4)	rrb = 0.59, p <0.0001	rrb = 0.38, n.s.	rrb = -0.37, p <0.0001
	Location Error (pixels)	227.5 (92.8)	105.9 (43.6)	85.9 (45.1)	rrb = -0.69, p <0.0001	rrb = -0.81, p <0.0001	rrb = -0.38, p <0.0001
	Location Error (z)	4.4 (3.4)	0.9 (1.7)	0.2 (1.4)	rrb = -0.64, p <0.0001	rrb = -0.77, p <0.0001	rrb = -0.33, p = 0.0002
	Identification Time (s)	4.1 (2.1)	2.6 (1.3)	2.4 (1.3)	rrb = -0.46, n.s.	rrb = -0.53, p <0.0001	rrb = -0.24, n.s.
	Identification Time (z)	3.0 (3.7)	0.7 (2.4)	0.3 (2.3)	rrb = -0.44, n.s.	rrb = -0.51, p <0.0001	rrb = -0.19, n.s.
	Localisation Time (s)	8.2 (3.5)	5.4 (2.1)	3.1 (1.5)	rrb = -0.49, p = 0.0007	rrb = -0.89, p <0.0001	rrb = -0.67, p <0.0001
	Localisation Time (z)	5.5 (4.6)	2.5 (2.7)	0.2 (1.4)	rrb = -0.43, n.s.	rrb = -0.87, p <0.0001	rrb = -0.63, p <0.0001
	Target Detection Rate (%)	53.1 (17.0)	76.2 (9.1)	69.2 (13.8)	rrb = 0.74, p <0.0001	rrb = 0.56, p <0.0001	rrb = -0.34, p = 0.0001
	Target Detection Rate (z)	-1.3 (1.6)	0.7 (0.9)	-0.1 (1.2)	rrb = 0.71, p <0.0001	rrb = 0.47, p = 0.0002	rrb = -0.46, p <0.0001
	Misbinding Error Rate (%)	36.9 (11.8)	21.7 (7.9)	28.1 (10.2)	rrb = -0.68, p <0.0001	rrb = -0.47, p = 0.0001	rrb = 0.42, p <0.0001
	Misbinding Error Rate(z)	0.7 (1.4)	-1.0 (0.9)	-0.1 (1.1)	rrb = -0.67, p <0.0001	rrb = -0.40, n.s.	rrb = 0.50, p <0.0001
	Guessing Error Rate (%)	28.4 (12.2)	13.0 (5.9)	16.7 (9.6)	rrb = -0.73, p <0.0001	rrb = -0.57, p <0.0001	rrb = 0.25, n.s.
	Guessing Error Rate (z)	1.6 (1.8)	-0.5 (0.9)	0.2 (1.3)	rrb = -0.71, p <0.0001	rrb = -0.49, p = 0.0001	rrb = 0.35, p <0.0001
	Imprecision (pixels)	133.3 (67.4)	67.4 (27.9)	54.2 (27.0)	rrb = -0.63, p <0.0001	rrb = -0.76, p <0.0001	rrb = -0.39, p <0.0001

	Imprecision (z)	5.1 (4.2)	1.4 (2.4)	0.2 (1.4)	rrb = -0.57, p = 0.0001	rrb = -0.76, p <0.0001	rrb = -0.44, p <0.0001
OIS	Object Identification Accuracy - Immediate Recall (%)	74.3 (16.1)	95.5 (5.3)	95.0 (7.5)	rrb = 0.87, p <0.0001	rrb = 0.83, p <0.0001	rrb = 0.05, n.s.
	Object Identification Accuracy - Immediate Recall (z)	-3.8 (3.2)	0.0 (1.2)	-0.3 (1.5)	rrb = 0.85, p <0.0001	rrb = 0.77, p <0.0001	rrb = -0.11, n.s.
	Object Identification Accuracy - Delayed Recall (%)	28.9 (19.9)	68.4 (18.2)	68.9 (18.7)	rrb = 0.83, p <0.0001	rrb = 0.84, p <0.0001	rrb = 0.01, n.s.
	Object Identification Accuracy - Delayed Recall (z)	-2.5 (1.3)	0.0 (1.2)	-0.1 (1.1)	rrb = 0.82, p <0.0001	rrb = 0.82, p <0.0001	rrb = -0.07, n.s.
	Semantic Identification Accuracy - Immediate Recall (%)	90.9 (10.8)	99.6 (1.5)	99.5 (3.4)	rrb = 0.64, p <0.0001	rrb = 0.65, p <0.0001	rrb = 0.06, n.s.
	Semantic Identification Accuracy - Immediate Recall (z)	-5.7 (4.6)	-0.6 (2.8)	-0.7 (4.0)	rrb = 0.62, n.s.	rrb = 0.75, n.s.	rrb = 0.34, n.s.
	Semantic Identification Accuracy - Delayed Recall (%)	38.9 (25.6)	79.6 (15.3)	79.4 (16.5)	rrb = 0.78, p <0.0001	rrb = 0.78, p <0.0001	rrb = 0.02, n.s.
	Semantic Identification Accuracy - Delayed Recall (z)	-3.1 (2.1)	-0.0 (1.1)	-0.1 (1.2)	rrb = 0.78, p <0.0001	rrb = 0.76, p <0.0001	rrb = -0.06, n.s.
	Location Error - Immediate Recall (cm)	3.2 (1.8)	1.0 (0.4)	1.9 (1.9)	rrb = -0.82, p <0.0001	rrb = -0.48, p <0.0001	rrb = 0.23, n.s.
	Location Error - Immediate Recall (z)	4.8 (5.5)	-0.1 (0.8)	0.2 (1.3)	rrb = -0.72, p <0.0001	rrb = -0.67, p <0.0001	rrb = 0.02, n.s.
	Location Error - Delayed Recall (cm)	5.3 (2.7)	1.9 (0.9)	2.3 (1.8)	rrb = -0.72, p <0.0001	rrb = -0.64, p <0.0001	rrb = 0.03, n.s.
	Location Error - Delayed Recall (z)	4.0 (3.5)	0.2 (1.0)	0.3 (1.5)	rrb = -0.70, p <0.0001	rrb = -0.68, p <0.0001	rrb = -0.05, n.s.
ROCF	Copy Raw Score (%)	78.5 (30.2)	98.3 (3.1)	97.3 (4.9)	rrb = 0.66, p <0.0001	rrb = 0.57, p <0.0001	rrb = -0.11, n.s.
	Copy Score (z)	-5.5 (8.8)	0.1 (0.9)	-0.3 (1.6)	rrb = 0.67, p <0.0001	rrb = 0.57, p <0.0001	rrb = -0.06, n.s.
	Immediate Recall Raw Score (%)	43.1 (22.8)	89.9 (11.8)	86.1 (13.2)	rrb = 0.92, p <0.0001	rrb = 0.88, p <0.0001	rrb = -0.17, n.s.
	Immediate Recall Score (z)	-4.4 (2.7)	0.3 (1.0)	-0.2 (1.3)	rrb = 0.89, p <0.0001	rrb = 0.83, p <0.0001	rrb = -0.29, p = 0.0007

	Mean Location Error	13.0 (5.1)	6.0 (2.3)	6.7 (4.7)	rrb = -0.83, p <0.0001	rrb = -0.72, p <0.0001	rrb = -0.06, n.s.
**CORSI**	Mean Location Error (z)	2.0 (1.7)	-0.2 (0.8)	0.3 (1.5)	rrb = -0.77, p <0.0001	rrb = -0.64, p <0.0001	rrb = 0.09, n.s.
	Number of Correct Responses	14.8 (7.0)	34.6 (9.3)	39.5 (12.3)	rrb = 0.89, p <0.0001	rrb = 0.93, p <0.0001	rrb = 0.22, n.s.
**DSST**	Number of Correct Responses (z)	-2.3 (1.1)	-0.2 (0.9)	-0.0 (1.2)	rrb = 0.86, p <0.0001	rrb = 0.87, p <0.0001	rrb = 0.10, n.s.
	TMT A Completion Time (s)	50.1 (26.5)	29.0 (11.0)	25.7 (8.2)	rrb = -0.79, p <0.0001	rrb = -0.86, p <0.0001	rrb = -0.23, n.s.
	TMT A Completion Time (z)	4.3 (5.3)	0.5 (1.8)	0.2 (1.3)	rrb = -0.79, p <0.0001	rrb = -0.82, p <0.0001	rrb = -0.12, n.s.
	TMT B Completion Time (s)	155.5 (150.9)	40.1 (11.4)	37.6 (13.1)	rrb = -0.88, p <0.0001	rrb = -0.90, p <0.0001	rrb = -0.16, n.s.
**TMT**	TMT B Completion Time (z)	13.8 (21.2)	0.3 (1.2)	0.1 (1.2)	rrb = -0.89, p <0.0001	rrb = -0.89, p <0.0001	rrb = -0.08, n.s.
	Average TMT Completion Time (s)	102.8 (87.8)	34.5 (9.9)	31.6 (9.5)	rrb = -0.89, p <0.0001	rrb = -0.92, p <0.0001	rrb = -0.19, n.s.
	Average TMT Completion Time (z)	10.3 (14.6)	0.4 (1.3)	0.1 (1.2)	rrb = -0.89, p <0.0001	rrb = -0.90, p <0.0001	rrb = -0.10, n.s.
	Motor speed (Time to connect two diagnal dots) (s)	6.5 (4.0)	3.8 (1.5)	4.4 (3.4)	rrb = -0.79, p <0.0001	rrb = -0.57, p <0.0001	rrb = -0.03, n.s.
	Motor speed (Time to connect two diagnal dots) (z)	2.2 (3.7)	0.0 (1.4)	0.3 (1.6)	rrb = -0.78, p <0.0001	rrb = -0.61, p <0.0001	rrb = 0.03, n.s.

Supplementary Methods 2 | Processing pipeline for plasma biomarkers

- 6 x10 ml blood in EDTA bottles was collected
- Within 30 minutes, blood was centrifuged at 1800 g for 10 minutes, at room temperature
- Plasma was then transferred into a 50 ml propylene tube and mixed
- It was then aliquoted into Fluid X cryovials (0.5 ml), scanned and logged
- Cryovials were kept in a fridge (4 C) for up to 8 hours
- They were then transferred into a -80 C freezer for long-term storage

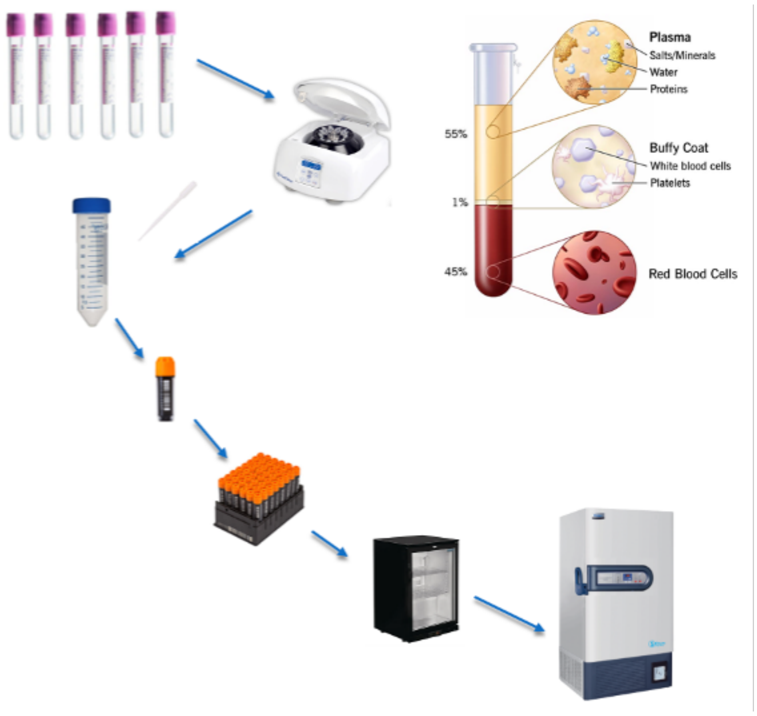

Plasma Aβ40, Aβ42, GFAP, and NfL were measured by Single molecule array (Simoa) technology using the Neurology 4-plexE assay on an HD-X analyser (Quanterix), according to manufacturer's instructions. Plasma pTau181 was also measured by Simoa using the pTau-181 Advantage assay on an HD-X analyser (Quanterix). Briefly, samples were thawed at 21°C, and centrifuged at 10,000 RCF for five minutes at 21°C. On-board the instrument, samples were diluted 1:4 with sample diluent and bound to paramagnetic beads coated with a capture antibody specific for human Aβ40, Aβ42, GFAP, NfL and pTau-181. Aβ40-, Aβ42-, GFAP-, NfL- and pTau181-bound beads were then incubated with a biotinylated anti-Aβ40, anti-Aβ42, anti-GFAP, anti-NfL and anti-pTau181 detection antibodies in turn conjugated to streptavidin-β-galactosidase complex. Subsequent hydrolysis reaction with a resorufin β-D-galactopyranoside substrate produces a fluorescent signal proportional to the concentration of Aβ40, Aβ42, GFAP, NfL and pTau181 present. Singlicate measurements were taken of each sample. Sample concentrations were extrapolated from a standard curve, fitted using a 4-parameter logistic algorithm. Intra-assay and inter-assay coefficients of variation (CVs) were less than 10% and 15% respectively, as determined by 8 quality controls following the same principles.

Supplementary Figure 1 | Group comparison for all digital cognitive metrics in Oxford Memory Task (OMT) and Object-in-Scene Task (OIS).

All metrics were normalised based on age-matched normative data.

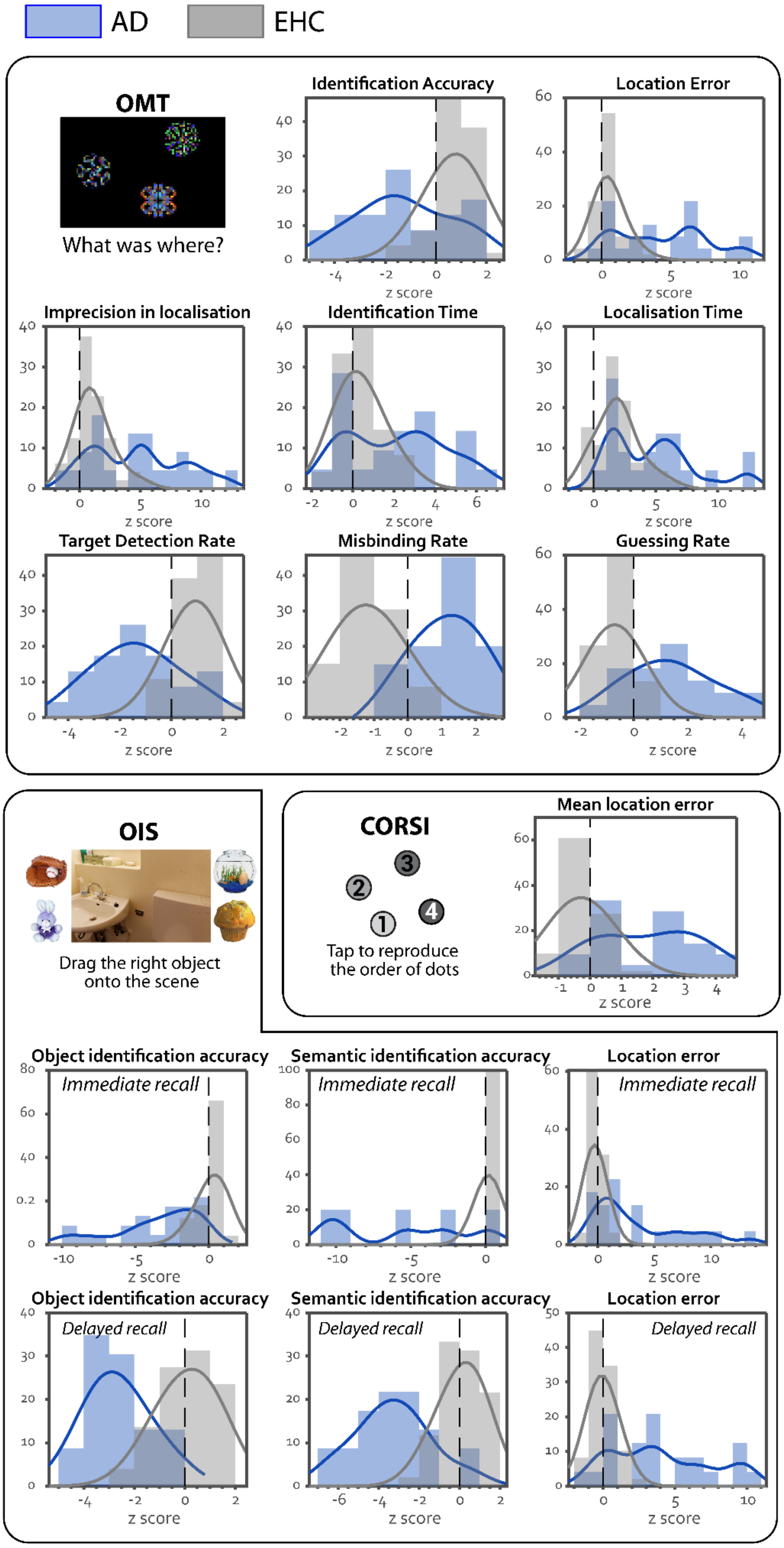

Supplementary Figure 2 | Group comparison for all digital cognitive metrics in ROCF, DSST and TMT.

ROCF = Rey–Osterrieth Complex Figure, DSST = Digit Symbol Substitution Task, TMT = Trail Making Test.

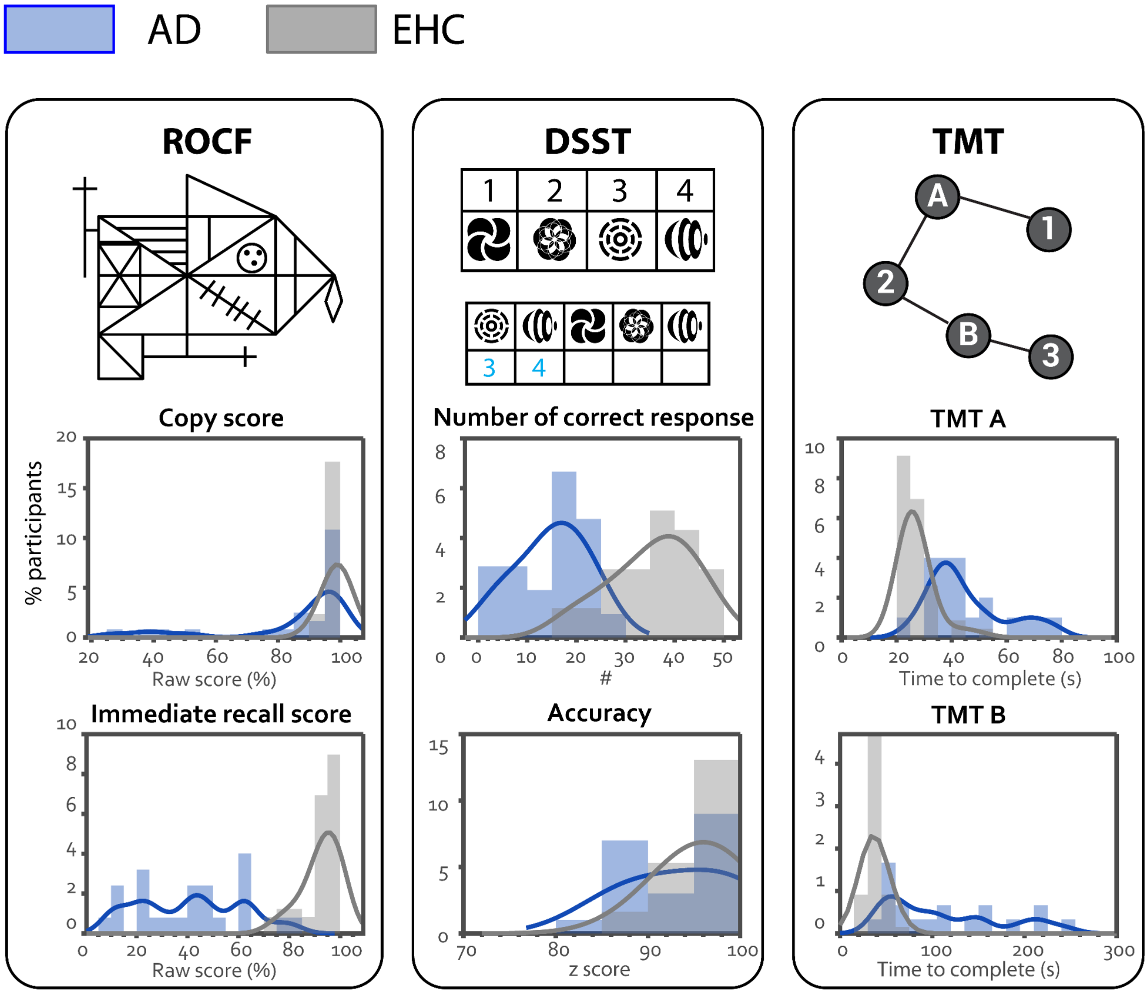

Supplementary Figure 3 | Within-group correlations between online digital cognitive tasks and plasma biomarkers.

Relationships between online digital cognitive tasks (x-axis) and plasma biomarkers (y-axis) in EHC1 (panel a) and AD (panel b). These results are uncorrected for multiple comparisons, as none of the correlations survived multiple comparisons correction. Significant results are highlighted by dark blue boxes. Significance is shown as: * p < 0.05, ** p < 0.01. Graded colors are used for directionality of the correlations (purple squares denote positive associations, coral squares negative associations).

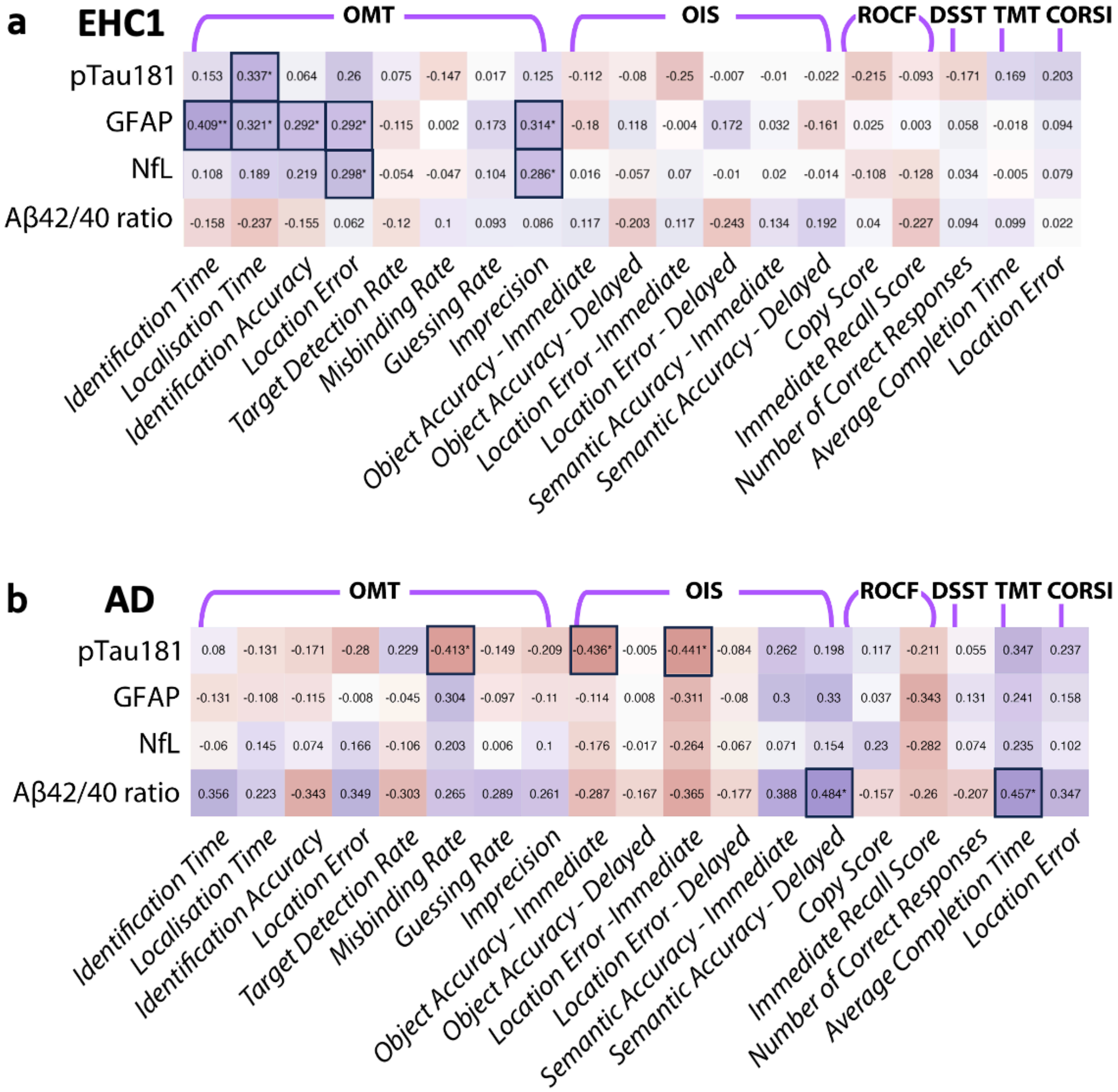

Supplementary Figure 4 | Which cognitive metric or plasma biomarker best predicts pTau181 or Aβ42/40 levels?

(a) Which cognitive metric or plasma biomarker best predicts pTau181? DSST was the winner. NfL and Aβ42/40 ratio significantly predict the level of pTau181, but ranked behind all digital tasks, with the exception of OMT. **(b) Ranked biomarkers and digital cognitive metrics in predicting Aβ42/40 ratio.** pTau181 was the only metric which significantly predicted Aβ42/40 ratio. OMT = Identification accuracy of the Oxford Memory Task, OIS = Object Identification Accuracy in Immediate Recall of the Object-in-Scene Memory Task, ROCF = recall of the Rey–Osterrieth Complex Figure, DSST = number of correct responses of the Digit Symbol Substitution Task, TMT = average reaction time of the Trail Making Test, CORSI = average location error of the Freestyle Corsi Block Task. **(c) Best combinations of biomarkers and digital cognitive metrics in predicting group (top), pTau181 (middle) and Aβ42/40 ratio (bottom).** AUC = area under the curve. AIC = Akaike information criterion.

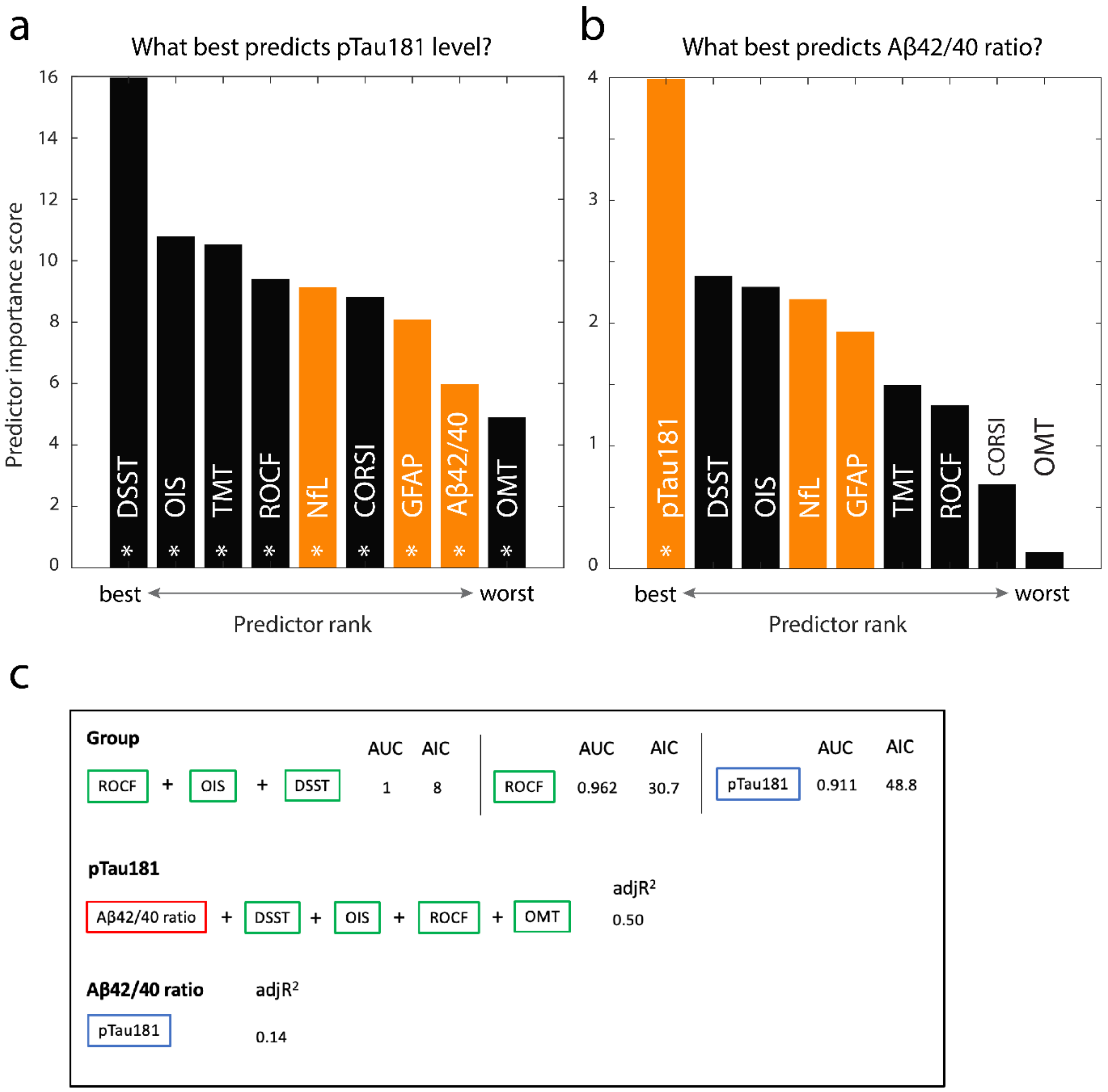

References for Supplementary Materials:

[1] Office for National Statistics. Ethnicity facts and figures of UK: Socioeconomic status 2020.

[2] Adler N, Stewart J. The MacArthur scale of subjective social status. MacArthur Research Network on SES & Health., San Francisco: 2007.

[3] Operario D, Adler NE, Williams DR. Subjective social status: reliability and predictive utility for global health. Psychology & Health 2004;19:237–46. https://doi.org/10.1080/08870440310001638098.

[4] Präg P, Mills MC, Wittek R. Subjective socioeconomic status and health in cross-national comparison. Social Science & Medicine 2016;149:84–92. https://doi.org/10.1016/j.socscimed.2015.11.044.
